# LTR retrotransposons transcribed in oocytes drive species-specific and heritable changes in DNA methylation

**DOI:** 10.1038/s41467-018-05841-x

**Published:** 2018-08-20

**Authors:** Julie Brind’Amour, Hisato Kobayashi, Julien Richard Albert, Kenjiro Shirane, Akihiko Sakashita, Asuka Kamio, Aaron Bogutz, Tasuku Koike, Mohammad M. Karimi, Louis Lefebvre, Tomohiro Kono, Matthew C. Lorincz

**Affiliations:** 1 Department of Medical Genetics, University of British Columbia, Vancouver, BC V6T 1Z3 Canada; 2 NODAI Genome Research Center, Tokyo University of Agriculture, Tokyo, 156-8502 Japan; 3 Department of BioScience, Tokyo University of Agriculture, Tokyo, 113-0033 Japan; 4 Present Address: Division of Reproductive Sciences, Cincinnati’s Children’s Hospital Medical Center, Cincinnati, OH 45229 USA; 5 Present Address: Department of Biological Sciences, Graduate School of Science, The University of Tokyo, Tokyo, 113-0033 Japan; 6 Present Address: MRC London Institute of Medical Sciences, Imperial College, London, W12 0NN UK

## Abstract

De novo DNA methylation (DNAme) during mouse oogenesis occurs within transcribed regions enriched for H3K36me3. As many oocyte transcripts originate in long terminal repeats (LTRs), which are heterogeneous even between closely related mammals, we examined whether species-specific LTR-initiated transcription units (LITs) shape the oocyte methylome. Here we identify thousands of syntenic regions in mouse, rat, and human that show divergent DNAme associated with private LITs, many of which initiate in lineage-specific LTR retrotransposons. Furthermore, CpG island (CGI) promoters methylated in mouse and/or rat, but not human oocytes, are embedded within rodent-specific LITs and vice versa. Notably, at a subset of such CGI promoters, DNAme persists on the maternal genome in fertilized and parthenogenetic mouse blastocysts or in human placenta, indicative of species-specific epigenetic inheritance. Polymorphic LITs are also responsible for disparate DNAme at promoter CGIs in distantly related mouse strains, revealing that LITs also promote intra-species divergence in CGI DNAme.

## Abstract (web summary)

De novo DNA methylation during mouse oogenesis occurs within transcribed regions. Here the authors investigate the role of species-specific long terminal repeats (LTRs)-initiated transcription units in regulating the oocyte methylome, identifying syntenic regions in mouse, rat and human with divergent DNA methylation associated with private LITs.

## Introduction

Long terminal repeat (LTR) retrotransposons, also known as endogenous retroviruses (ERVs), constitute ~10 and ~8% of the mouse and human genome, respectively^1^. While their expression is generally suppressed by DNAme and/or repressive histone modifications^2^, a subset of ERV subfamilies retain transcriptional activity in specific cell/tissue types^3^. ERVs are especially active in germ cells and early embryos^4^, coinciding with the expression of numerous tissue-specific LTR-driven chimeric transcripts^5–7^. Indeed, over 15% of all transcripts initiate in an LTR in mouse oocytes^5,6,8^, most in mammalian apparent LTR retrotransposons (MaLRs), which constitute ~5% of the genome^1^. Members of the mouse transcript (MT) subfamily^9^ of MaLRs are particularly active in oocytes and hundreds of MT LTRs have been co-opted as oocyte-specific gene promoters^5,6^. For example, an intragenic MTC element in the *Dicer* gene produces an alternative transcript in mouse oocytes that encodes DICER1o, a truncated but hyperactive isoform of the protein^10^. Notably, while ancestral MT elements colonized the common rodent ancestor of the mouse, rat, and naked mole rat^6^, this family is absent from the primate lineage^9^. Conversely, human oocytes also harbor a significant number of transcripts that initiate in LTRs, including of the distantly related THE1 MaLR family, which is absent from the rodent lineage^9^.

Following global erasure in primordial germ cells (PGCs), DNAme is re-established postnatally in association with transcribed regions in mouse and human oocytes^8,11,12^. As genic H3K36me3 deposition, including at intragenic CpG islands (CGIs), precedes DNMT3A/3L-dependent de novo DNAme in mouse oocytes^11,13^, this histone mark likely guides de novo DNAme during oogenesis. Indeed, H3K36me3, which is deposited by SETD2 in association with the RNA polII machinery, plays a critical role in promoting DNMT3B-dependent gene body DNAme in mouse embryonic stem cells^14^. Furthermore, several hundred CGIs embedded within oocyte-specific transcripts, a subset initiating in an ERV, are clearly de novo DNA methylated during mouse oogenesis^8^.

As LTR retrotransposons are highly variable across species, with thousands of annotated elements in mice absent from orthologous positions in rat or human and vice versa, we examined the impact of LTRs on species-specific transcription and the establishment of DNAme in mammalian oocytes. Comparing mouse, rat, and human, we identify hundreds of species-specific genic and intergenic transcripts, many initiating in solo LTRs private to a single species. These LTR-initiated transcription units (LITs) are associated with domains of species-specific DNAme, which in mouse oocytes coincide with transcription-coupled H3K36me3 deposition. Furthermore, methylation at a subset of CGI promoters embedded within such LITs persists on the maternal allele at least through the blastocyst stage in mice or extraembryonic tissues in human. Finally, we show that LTRs polymorphic between two distantly related mouse strains likely promote strain-specific DNAme states in oocytes, including at CGI promoters.

## Results

### Divergent intergenic DNAme in mammalian oocytes

**Fig. 1:**
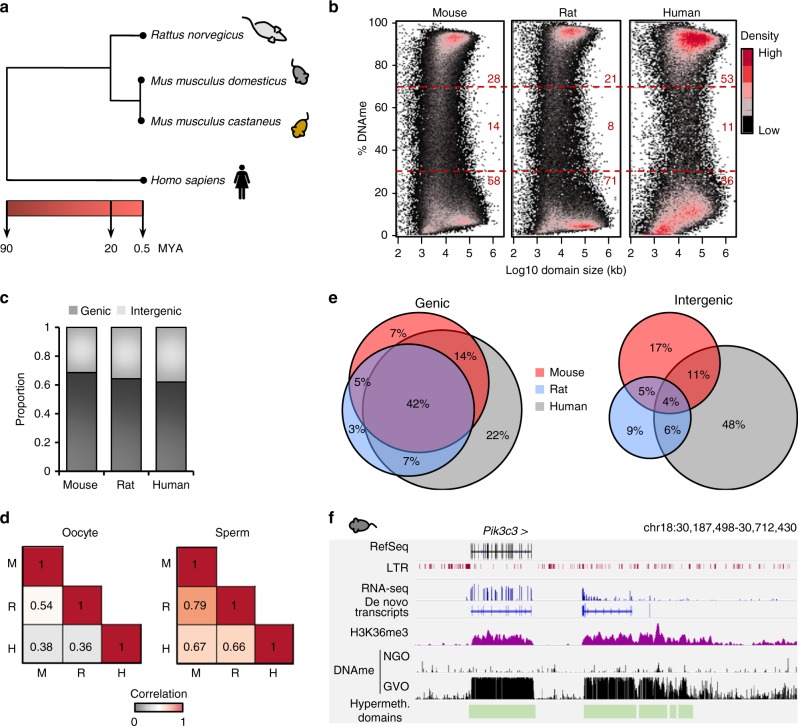
Syntenic intergenic regions show divergent DNA methylation in rodent and human oocytes. **a** Evolutionary distance between rat, human, and the two mouse strains used in this study (adapted from http://timetreebeta.igem.temple.edu/). **b** Density plots depicting the distribution of DNAme in mouse, rat, or human oocytes. The percentage of each genome with low (<30%) or high (>70%) DNAme is indicated in red. DNAme domains were identified using Changepoint analysis. **c** Proportion of hypermethylated (>70% DNAme) genome-wide 1 kb bins in genic or intergenic regions. Total number of bins with sufficient coverage: mouse: 934,621 genic/1,132,257 intergenic; rat: 794,069 genic/1,195,429 intergenic; human: 1,024,005 genic/1,132,257 intergenic. **d** Heat map of the correlation between DNAme patterns in oocytes or sperm over 433,111 syntenic genomic regions (1 kb bins with >5× coverage over >5 CpGs in all 3 species). **e** Venn diagrams showing the overlap in hypermethylated (>70% DNAme) syntenic genomic regions (1 kb bins). Methylated bins in syntenic intergenic regions are more divergent than those overlapping an annotated gene (mm10, rn6, or hg19 Ensembl annotation) in all three species. **f** Genome browser screenshot of the *Pik3c3* locus in mouse oocytes. Note the presence of de novo DNAme and H3K36me3 coincident with the predicted intergenic transcription units. Mouse and human WGBS datasets (described in the data summary table) are from refs. ^11,12,16^

To study the conservation of DNAme and transcription in mammalian oocytes, we focused on mouse, rat, and human, which are separated by 20 and 90 million years of evolution, respectively (Fig. 1a). We generated whole-genome bisulfite sequencing (WGBS) libraries from rat oocytes and sperm using the post-bisulfite adaptor tagging (PBAT) method^15^ and compared these data to published mouse^11,16^ and human^12^ libraries. As observed in mouse and human, DNAme has a bimodal distribution in rat oocytes (Fig. 1b), with most of the genome either hypermethylated (>70% DNAme) or hypomethylated (<30% DNAme). However, hypermethylated domains in rat oocytes encompass only 21% of the genome, vs. 28% in mouse and 53% in human (Fig. 1b). In contrast, overall DNAme levels are far more concordant in sperm, with an average of 90%, 94%, and 87% DNAme in mouse, rat, and human, respectively (Supplementary Figure 1a).

DNAme in rat oocytes is generally found in gene bodies, as reported in mouse and human oocytes, where this mark is positively correlated with active transcription^8,11,12,16^. To compare the relationship between gene transcription and gene body DNAme across species, we generated total RNA-seq libraries from mouse and rat oocytes and analyzed these datasets in parallel with recently published human oocyte RNA-seq data^17^. As in mouse and human, we find that DNAme is positively correlated with genic transcription in rat oocytes, with 85%, 91%, and 82% of transcribed genes (>1 FPKM (fragments per kilobase per million mapped sequence reads)) harboring >40% gene body DNAme, respectively (Supplementary Figure 1b-e). In all three species, however, ~1/3 of hypermethylated regions are intergenic (Fig. 1c and Supplementary Table 1). To identify common and species-specific hypermethylated intergenic domains, we compared mouse, rat, and human oocyte or sperm DNAme in syntenic 1 kb genomic bins (Fig. 1d and Supplementary Table 2). As anticipated, murine (mouse and rat) oocyte methylomes show greater overall concordance to each other than to human oocytes and >40% of all syntenic genic regions methylated in at least one species are methylated in all three species (Fig. 1e). In contrast, hypermethylated regions overlapping a syntenic intergenic region in at least one species are far less likely to be methylated in all three species, implicating relatively high levels of lineage-specific intergenic transcription.

To corroborate the identification of intergenic transcribed domains in mouse oocytes, we generated high-resolution ULI-NChIP^18^ libraries for H3K36me3. While this mark is generally enriched over actively transcribed, hypermethylated gene bodies (Fig. 1f and Supplementary Figure 1b), as expected, enrichment is also observed over hypermethylated intergenic regions, including syntenic regions hypomethylated in rat and/or human oocytes (Fig. 1f and Supplementary Figure 1g). Taken together, these observations reveal that inter-species differences in DNAme are much higher in intergenic than genic regions and that gene bodies and intergenic regions likely share a common mechanism of transcription-coupled de novo DNAme in mammalian oocytes.

### LTR-initiated transcription impacts de novo DNAme in oocytes

LTR transcription was previously shown to be a hallmark of mouse^5,6,8^ and, to a lesser extent, human oocytes^6,17^. As oocytes exhibit more species-specific DNAme patterns in intergenic regions (Fig. 1e and Supplementary Figure 1f), and LTR elements insertion sites are highly variable both between and within species^19^, we investigated the contribution of LITs to inter-species DNAme divergence. For each species, we used the LIONS pipeline^20^, which exploits CuffLinks de novo transcriptome assembly^21^, to identify all transcripts overlapping with an annotated repetitive sequence. Focusing our attention on transcripts initiating in an ERV (annotated as an LTR or internal region), we identified 3384 LITs in mouse, 1494 in rat, and 1056 in human oocytes (Supplementary data 1-3).

**Fig. 2:**
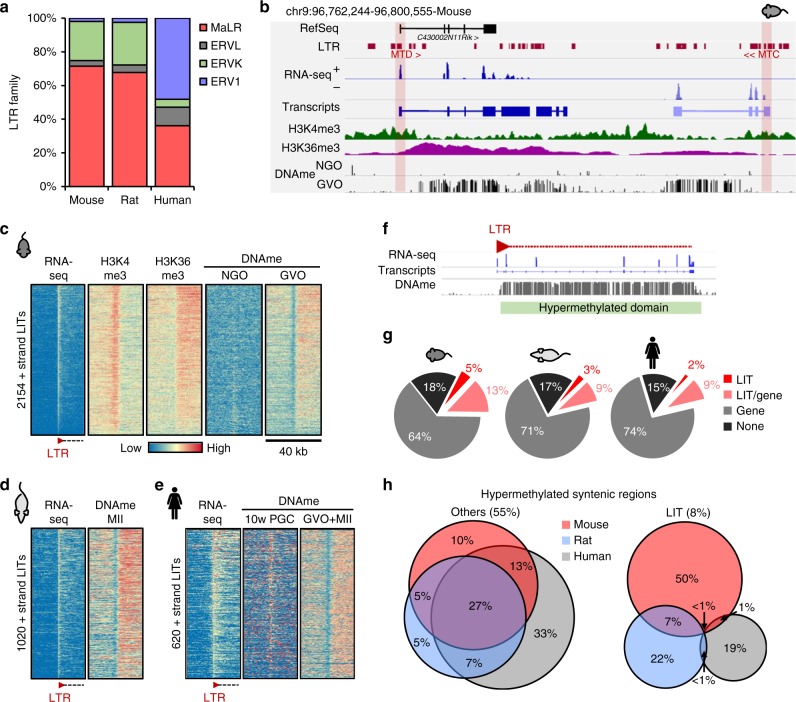
LTR-initiated transcription impacts species-specific DNAme in oocytes. **a** Bar chart of the relative contribution of LTR retrotransposon classes driving all LITs in mouse, rat, and human oocytes. **b** Screenshot of genic and intergenic LITs resulting in two hypermethylated domains in mouse oocytes: the canonical TSS of the *C430002N11Rik* gene is embedded in an MTD, and a second intergenic transcript initiates in an MTC. Both LTR promoters are enriched for H3K4me3 while the downstream de novo methylated regions are enriched for H3K36me3 in GVO. **c**–**e** Heat maps of the correlation between transcription and downstream (+strand) DNAme at 2154 LITs (TSS ±20 kb) in mouse oocytes, 1020 LITs in rat oocytes, and 620 forward strand LITs in human oocytes. NGO non-growing oocytes, GVO germinal vesicle oocytes, MII metaphase II oocytes, 10 wk PGCs 10-week female PGCs (human). **f** Cartoon illustrating a DNA methylated genomic region that is overlapped by an LIT. **g** Pie charts showing the proportion of hypermethylated regions (normalized to the domain size) that are overlapped by an LIT and/or an annotated Ensembl gene in mouse, rat, or human oocytes. **h** Venn diagrams showing syntenic 1 kb bins that are hypermethylated (>70% DNAme) in mouse, rat, and/or human oocytes. Syntenic regions that overlap with an LIT (right) show significantly greater divergence in DNAme between species than the rest of the genome (left). The percentage of total syntenic genomic regions (433, 111 1 kb bins with >5 CpGs with >5× coverage) is indicated above each Venn diagram. Mouse and human WGBS datasets analyzed from refs. ^11,12,16,23^, human RNA-seq data from ref. ^17^, and mouse H3K4me3 from ref. ^24^

The majority of LITs identified in mouse (72%) and rat (68%) oocytes initiate in a MaLR (Fig. 2a), predominantly of the MT superfamily, which is restricted to the rodent lineage (Supplementary Figure 2a, b). Furthermore, >40% of mouse oocyte LITs originate in LTRs of the most recently inserted MT subfamily MTA, which is absent from the rat genome. Consistent with previous observations^6^, we find that MaLRs are not as transcriptionally active in human oocytes, accounting for only 36% of LITs (Fig. 2a), with most MaLR initiation events occurring in either THE1 or MLT1 elements (Supplementary Figure 2a). Murine oocytes also exhibit a greater fraction of transcripts initiating in ERVK elements than human oocytes (~25% vs. 5% of LITs, respectively; Fig. 2a and Supplementary Figure 2a), perhaps due to their significantly greater abundance in rodent genomes^22^. Conversely, primates carry significantly more ERV1 copies than rodents, and nearly half of LITs in human oocytes initiate in the LTRs of ERV1 elements, such as LTR12.

Consistent with the pattern of DNAme over actively transcribed genes (Supplementary Figure 1b-e), DNAme is detected downstream of putative LIT transcription start sites (TSSs) in mouse, rat, and human oocytes (Fig. 2b–e). The same regions are hypomethylated in mouse non-growing oocytes (NGOs) and human female PGCs^23^, consistent with transcription-coupled de novo DNAme during oocyte growth. Furthermore, H3K4me3^24^ and H3K36me3 are enriched directly over and downstream, respectively, of these putative LIT TSSs in mouse germinal vesicle oocytes (GVOs) (Fig. 2b, c), further supporting the model that de novo DNAme downstream of active LTR elements in oocytes is transcription-coupled.

To study the impact of LTR-initiated transcription on DNAme in mammalian oocytes in greater detail, we identified hypermethylated genomic regions that overlap with an LIT and/or an annotated gene in each species (Fig. 2f, g). Consistent with the greater number of LITs identified in mice, a higher fraction of hypermethylated regions appear to result from LTR-initiated transcription in this species (18%) than in rat (12%) or human (11%) oocytes. Over 63% of regions syntenic between the three species show >70% DNAme in at least one species, including 8% apparently resulting from an LIT (Fig. 2h). Intriguingly, hypermethylated regions overlapping with an LIT are far more likely to exhibit species-specific DNAme, with 50% of hypermethylated regions (totaling ~4% of syntenic genomic regions; Supplementary Table 2) associated with an LIT unique to mouse. In all three species, however, a significant percentage of hypermethylated domains (15–18%) do not overlap with either an annotated gene or an LIT, implicating oocyte-specific non-repetitive promoters and/or the presence of additional LTR-initiated transcripts that are not detected by de novo transcriptome assembly.

As de novo transcriptome assembly is biased toward highly expressed regions and can generate fragmented transcripts, we are likely underestimating the number of LITs. We therefore estimated the total number of active LTR TSSs by identifying all annotated ERVs with transcript levels >1 FPKM in each species. Consistent with the analyses described above, mouse oocytes display the highest number of active LTR elements, with over 12,157, compared with 7487 and 9059 in rat and human oocytes, respectively (Supplementary Figure 2c). Of note, MaLR transcription is over-represented relative to the genomic abundance of this category of elements in mouse and rat oocytes, while ERV1 transcription is over-represented compared to its genomic abundance in human oocytes (Supplementary Fig 2d). Consistent with the LITs identified above, we observe de novo DNAme downstream of these transcriptionally active solo LTRs in murine oocytes (Supplementary Figure 2e-f). On the other hand, consistent with the higher level of global DNAme in human oocytes, hypermethylation is observed both upstream and downstream of solo LTRs, making it more challenging to visualize the relationship between LITs and de novo DNAme in this species (Supplementary Figure 2g). Nevertheless, these data indicate that a significant fraction of transcripts in mammalian oocytes initiate in an LTR element and reveal that regions downstream of such LTR TSSs are generally de novo DNA methylated in oocytes.

### Impact of LITs on oocyte gene expression and DNAme

**Fig. 3:**
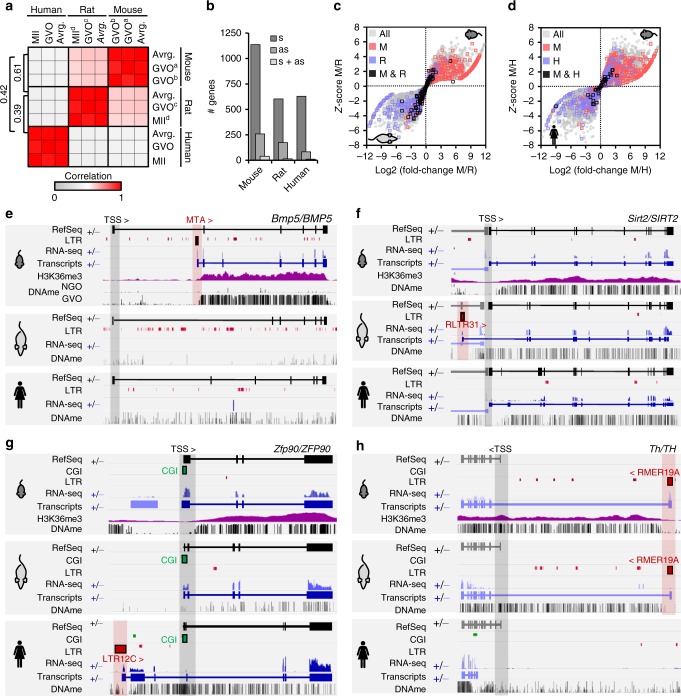
LTR-initiated transcription impacts species-specific gene transcription and gene body DNAme in oocytes. **a** Pearson correlation between mouse, rat, and human oocyte transcript levels over 11,186 syntenic Ensembl annotated genes. ^a^C57BL/6, ^b^Cast/Ei, ^c^Sprague Dawley, ^d^Wistar Han. **b** Number of annotated Ensembl genes with one or more overlapping LIT(s). s: sense overlap, splicing into a genic exon and >10% contribution to all of the gene’s isoforms, as antisense overlap. **c** Comparison of gene transcription between mouse and rat GVOs (gray). Highlighted are genes with an LTR-driven isoform that contributes to at least 10% of the gene transcripts in mouse (red), rat (blue), or in both species (black). **d** Comparison of gene transcription between mouse and human GVOs (gray). Highlighted are genes with an LTR-driven isoform that contributes to at least 10% of the gene transcripts in mouse (red), human (blue), or in both species (black). **e** Screenshot of the *Bmp5/BMP5* locus. In mouse oocytes, transcription initiates from an intragenic MTA element, and gene body H3K36me3 and DNAme are observed only downstream of the MTA. Note that the ortholog is not transcribed in rat or human oocytes. **f** Screenshot of the *Sirt2/SIRT2* locus. In rat oocytes, transcription initiates in an upstream RLTR31B2 element, and DNAme downstream of this alternative promoter encompasses the canonical *Sirt2* promoter. In both mouse and human oocytes, the orthologous gene is transcribed from the canonical (hypomethylated) promoter. **g** Screenshot of the *Zfp90/ZFP90* locus. A human-specific isoform initiates in an LTR12C upstream of the canonical CGI promoter, which is hypermethylated exclusively in human oocytes. *Zfp90* is transcribed from the canonical TSS in mouse and rat oocytes. **h** Screenshot of the *Th* locus showing an unannotated isoform that initiates in an RMER19A LTR- upstream of the canonical promoter exclusively in mouse and rat oocytes. DNAme extends downstream of the RMER19A TSS in both species, coincident with H3K36me3 in mouse, and encompasses the annotated *Th* TSS. Mouse and human WGBS datasets analyzed from refs. ^12,16^ and human RNA-seq data from ref. ^17^

As LIT-associated hypermethylated domains frequently encompass annotated genes (Fig. 2g), including their promoter regions, we wished to determine how LTR-initiated transcription impacts species-specific gene expression. To compare oocyte transcriptomes across species, we generated total RNA-seq datasets from mouse inbred strains C57BL/6 and Cast/Ei and rat strains Wistar Han and Sprague-Dawley and compared these to recently published human oocytes RNA-seq datasets^17^ (Fig. 3a). As anticipated, oocyte transcriptomes within the same species show the highest correlation over syntenic genes while inter-species expression profiles are most similar between mouse and rat.

From the lists of LITs generated above, we identified those with sense or antisense overlap with an annotated gene, as either may affect overall genic transcript levels. Notably, nearly twice as many genes in mouse oocytes (1176 genes) had an LTR-initiated isoform splicing into an annotated genic exon (sense) than in rat or human oocytes (617 and 639 genes, respectively) (Fig. 3b). Over 75% of such chimeric transcripts in rodent oocytes initiate in a MaLR element, a subset of which encode a canonical splice donor within the consensus LTR sequence^4–6^. Comparison between mouse and rat or mouse and human oocytes reveals that transcription from an alternative LTR TSS unique to one species is frequently associated with elevated transcript levels of the cognate gene relative to the syntenic gene in the other species (Fig. 3c, d and Supplementary Figure 3a). For example, transcription of the mouse *Bmp5* gene initiates in an intragenic MTA element, which then splices into exon 2 generating a chimeric transcript (Fig. 3e). Consistent with the absence of MTA elements in the rat and human genomes, however, *Bmp5/BMP5* transcripts are absent in the oocytes of these species, and only mouse oocytes gain DNAme downstream of this alternative TSS.

Remarkably, analysis of species-specific chimeric transcripts initiating upstream of an annotated gene reveals that DNAme is gained over the canonical genic TSS. For example, DNAme of the promoters of *Sirt2* in rat (Fig. 3f) and *Dnmt3b* in mouse (Supplementary Figure 3b) is apparently gained as a consequence of LITs initiating in upstream RLTR31B2 and MTA LTRs, respectively. Similarly, DNAme of the promoters of human *ZFP90* and *SCIN* likely results from chimeras that initiate in upstream LTR12C elements, which are primate-specific (Fig. 3g and Supplementary Figure 3c). Finally, murine-specific chimeric transcripts, such as an RMER19-initiated transcript that splices into the *Th* gene (Fig. 3h), promote DNAme of the genic TSS in both mouse and rat oocytes, whereas the canonical human *TH* promoter remains hypomethylated.

### LITs promote species-specific CGI methylation in oocytes

**Fig. 4:**
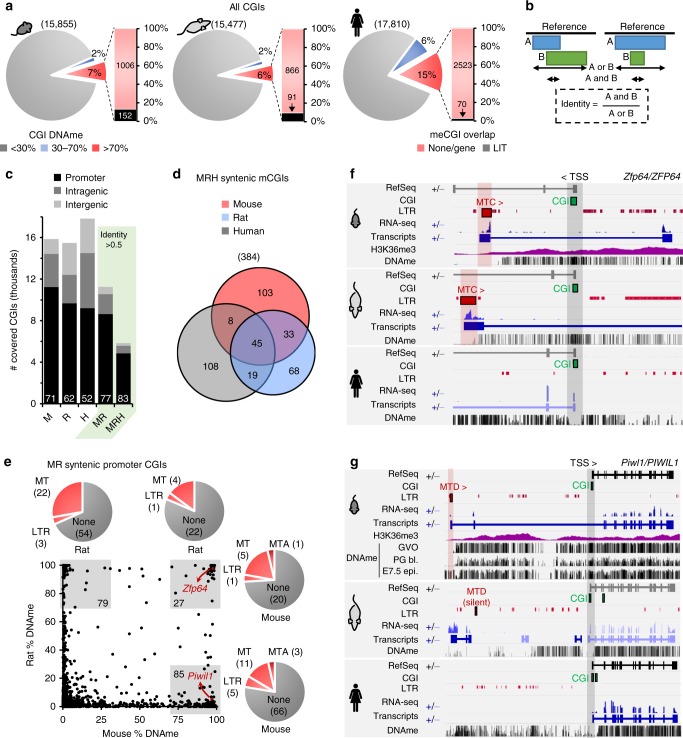
LTR transcription leads to species- and rodent-specific CpG island (CGI) methylation. **a** Proportion of CGIs (>5 CpGs covered by WGBS) with low (<30%), intermediate (30–70%), or high (>70%) DNAme levels in mouse, rat, and human oocytes. The proportion and number of hypermethylated CGIs embedded within an LIT is depicted in the accompanying bar chart (black). **b** Identification of syntenic CGIs between two species by calculation of identity. CGIs with an identity >0.5 between two species were included in our analyses. **c** Proportion of CGIs overlapping with an annotated TSS (±500 bp; promoter CGIs), gene body (intragenic), or intergenic region in human (H), mouse (M), rat (R), mouse + rat (MR), and mouse + rat + human (MRH). Total number of CGIs with >5 CpGs >5× WGBS coverage in each species and subset of CGIs syntenic (identity >0.5) between mouse (M) and rat (R) or all three species are shown. The percentage of CGIs overlapping a TSS is also shown for each. **d** Venn diagram showing the overlap in DNAme at all syntenic CGIs hypermethylated in in at least one of mouse, rat, or human oocytes (384). **e** Syntenic promoter CGIs hypermethylated in mouse and/or in rat oocytes (gray boxes). The proportion of meCGIs that overlap with a transcript initiated in an MTA, a non-MTA MT element (MTB, MTC, MTD, or MTE), or a non-MT LTR element is depicted in the adjacent pie charts. **f** Genome browser screenshots of the *Zfp64* CGI promoter. In mouse and rat oocytes, an MTC-driven antisense LIT overlapping the canonical promoter CGI appears to be responsible for DNAme, consistent with the H3K36me3 profile in mouse oocytes. **g** Screenshot of the *Piwil1* locus illustrating transcription and DNAme in mouse, rat, and human oocytes. H3K36me3 in oocytes and DNAme in PG blastocysts and E7.5 embryos are also shown for mouse. A mouse-specific *Piwil1* LIT initiates in an MTD element situated upstream of the canonical TSS, and only the mouse CGI promoter is hypermethylated. Mouse and human WGBS datasets analyzed from refs. ^12,16^ and human RNA-seq data from ref. ^17^

Nearly 10% of CGIs are de novo DNA methylated in mouse oocytes, ~30% of which map either to promoter or intergenic regions^11^. To determine whether a subset of such methylated CGIs (meCGIs) are embedded within LITs, we first measured DNAme levels over all annotated CGIs, which are generally hypomethylated in each species (Fig. 4a). Notably, 7%, 6%, and 15% of CGIs show >70% DNAme in mouse, rat, and human oocytes, respectively, with ~2% of promoter CGIs hypermethylated in each species (Supplementary Figure 4a). As previously observed in mice^11^ and humans^12^, DNAme of promoter CGIs in rats is far more prevalent in oocytes than in sperm (Supplementary Figure 4b), despite the overall higher level of DNAme in the latter. Indeed, over 95% and 90% of CGIs hypermethylated in oocytes are hypomethylated in rodent and human sperm, respectively, revealing that widespread CGI hypermethylation may generally be restricted to the female germline in mammals.

To determine the extent to which such CGI methylation might be explained by LTR-initiated transcription in oocytes, we identified all meCGIs that are embedded within an LIT in each species. The highest percentage of such meCGIs is found in mouse oocytes, with 13.1% (152/1158) overlapping an LIT, followed by 9.5% (91/957) in rat oocytes and only 2.7% (70/2593) in human oocytes (Fig. 4a). Interestingly, in all species, an even higher proportion of promoter meCGIs overlap with an LIT, with 55 (22.0%), 39 (19.3%), and 15 (7.6%) detected in mouse, rat, and human oocytes, respectively (Supplementary Figure 4a). Furthermore, nearly all promoter CGIs hypermethylated as the apparent result of LTR-initiated transcription in oocytes are hypomethylated in sperm of the same species (Supplementary Figure 4b).

If such CGI hypermethylation is orchestrated by transcription initiating in LTRs, then syntenic CGIs should be hypomethylated in species lacking the cognate LTR. To evaluate the divergence of CGI DNAme between species, we measured CGI identity between the mm10, rn6, and hg19 annotations (Fig. 4b). Genic regions, particularly around gene promoters, show higher conservation across species, and as such, a higher proportion of promoter CGIs can be identified as syntenic between mouse/rat or mouse/rat/human annotations than non-promoter CGIs (Fig. 4c). As anticipated, mouse and rat oocytes share a greater number of syntenic meCGI than are shared between either species and human oocytes (Fig. 4d). Of the 384 syntenic CGIs that are hypermethylated in at least one species, 45 are common between all 3 species, 39 of which are intragenic (>500 bp from the TSS).

To further evaluate the role of LTR-initiated transcription in DNAme of promoter CGIs, we focused on CGIs syntenic between mouse and rat, nearly doubling the number of CGIs that could be evaluated (Fig. 4c). Intriguingly, most promoter meCGIs in mouse and/or rat oocytes are hypomethylated in the alternative species, with 85 mouse-specific and 79 rat-specific meCGIs, vs. only 27 shared (Fig. 4e). Furthermore, a significant proportion of the private and shared promoter meCGIs appear to be the result of LITs, many initiating in MT elements. A few of such transcripts private to mouse oocytes initiate in an MTA, a subfamily unique to this species. The canonical *Dnmt3b* CGI promoter, for example, is hypermethylated only in mouse as the result of transcription initiating in an upstream MTA LTR (Supplementary Figure 3b). Moreover, most LTRs upstream of meCGIs shared between mouse and rat oocytes are annotated as MTB, MTC, or MTD, which are found in both species. For example, an antisense LIT initiating in an orthologous intragenic MTC element in the *Zfp64* gene encompasses the annotated genic CGI promoter, which is hypermethylated in both mouse and rat oocytes (Fig. 4f).

Curiously, however, several of the LITs that encompass CGIs hypermethylated only in mice initiate within LTRs of MTB, MTC, or MTD MaLR families. For example, *Piwil1*, which encodes an argonaute protein involved in piRNA biogenesis, is transcribed from an MTD ~33 kb upstream of the canonical TSS in mouse oocytes, apparently resulting in DNAme over the CGI promoter (Fig. 4g). Though no RNA-seq coverage is detected over exon 1 of the canonical gene, the annotated start codon is located in the third exon, which is retained in the chimeric transcript, and *Piwil1* is expressed in growing mouse oocytes^25^. While an annotated MTD element is present ~28 kb upstream of the rat *Piwil1* promoter, this CGI remains hypomethylated. This paradoxical observation is likely explained by the fact that, unlike in the mouse, transcripts initiating in this upstream MTD are not detected in the rat (Fig. 4g), while RNA-seq coverage is clearly detected over exon 1 of the annotated rat *Piwil1* gene. Comparison of the sequence of this MTD and its likely ortholog in the mouse reveals several sequence differences that may adversely affect its transcriptional activity in rat oocytes (Supplementary Figure 4c). Taken together, these data indicate that species-specific CGI hypermethylation can arise as a consequence of transcription initiating in a species-specific LTR element or in a shared LTR that has lost transcriptional competency in one species.

### Persistence of LIT-associated maternal DNAme post-fertilization

**Fig. 5:**
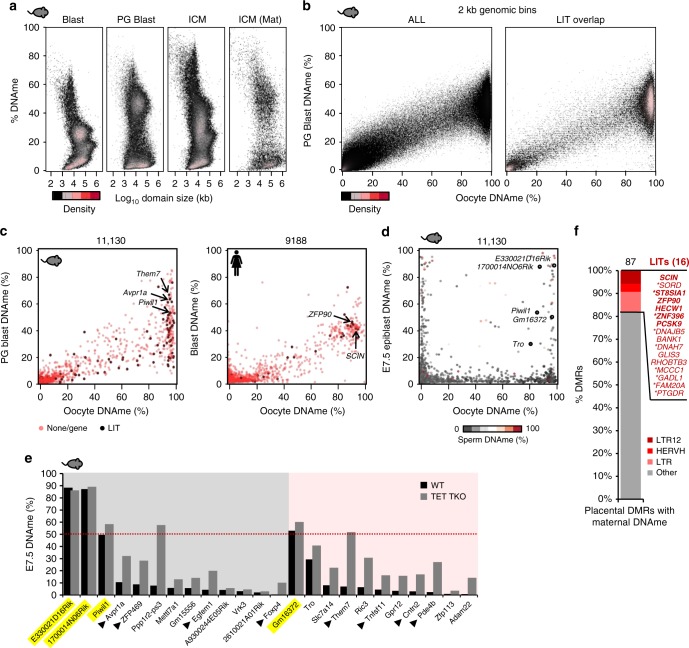
Persistence of LIT-associated oocyte DNAme in mouse and human following fertilization. **a** Density plot depicting the distribution of DNAme in mouse blastocyst, PG blastocyst, ICM (C57BL/6×DBA F1 cross) and ICM (maternal allele). DNAme domains were identified using Changepoint analysis. **b** Density plots comparing oocyte DNAme to PG blastocysts over genome-wide 2 kb bins (left) or 2 kb bins overlapping an LIT (right). **c**. Scatter plots of oocyte vs PG blastocyst (mouse) or blastocyst (human) CGI promoter DNAme. Promoter CGIs overlapped by an LIT in oocytes are highlighted in black. **d** Scatter plots of oocyte vs E7.5 epiblast CGI promoter DNAme. Promoter CGIs overlapped by an LIT in oocytes are highlighted in black. Sperm DNAme is indicated as a color gradient. Note that the rat *Piwil1* gene is oriented in the reverse direction. **e** DNAme in WT or *Tet* TKO E7.5 embryos over 24 promoter CGIs that gain DNAme as the result of LTR-initiated transcription in oocytes. Yellow highlight: genes showing apparent retention of maternal DNAme in E7.5 embryos. Black arrow: genes identified as TET targets in Dai et al.^28^). Gray background: LITs present in C57BL/6 and Cast/Ei meCGIs; pink background: C57BL/6 private LITs. Note that the genes *E330021D16Rik* and *1700014NO6Rik* also gain DNAme on the paternal genome in the peri-implantation stage (see **d**). **f** Bar chart including 87 CGIs methylated on the maternal allele in human placenta^29^, categorized here via LIONS or manual inspection of de novo transcripts (*), as LIT or non-LIT (other) associated in oocytes. The 16 LIT-associated DMRs are further subcategorized based on annotated 5′ LTR. Bold: DMRs associated with paternal-specific gene transcription in placenta (ref. ^30^). Mouse and human WGBS datasets analyzed from refs. ^11,12,16,23,26^

To determine whether such LTR-driven de novo DNAme persists on the maternal genome following fertilization, we generated PBAT libraries from parthenogenetic (PG) mouse blastocysts. Consistent with previous reports showing substantial retention of DNAme on the maternal allele following DNAme reprogramming in the early embryo^26^, relatively high methylation levels are evident in PG blastocysts (which harbor genomic DNA exclusively of maternal origin), as well as on the maternal genome in the inner cell mass ICM of F1 mice (Fig. 5a and Supplementary Figure 5a, b). Hypermethylated regions overlapping with LITs reveal a level of DNAme retention in PG blastocysts similar to that of all genomic regions (Fig. 5b), indicating that DNAme resulting from LTR transcription in the oocyte can be transmitted to the progeny.

Many meCGI promoters in mouse oocytes, including the majority of those embedded in LITs, such as *Piwil1*, *Avpr1a*, and *Them7*, also show persistence of DNAme in PG blastocysts (Fig. 5c). However, most of these LIT-associated meCGI promoters become hypomethylated by E7.5 (Fig. 5d), likely as a result of TET1 activity in the early post-implantation embryo^27^. Indeed, eight of these CGIs, including *Avpr1a* and *Them7*, show increased methylation in *Tet1/Tet2/Tet3* triple-knockout E7.5 epiblasts^28^ (Fig. 5e). Nevertheless, several oocyte-specific meCGIs, including the promoter regions of *Piwil1*, *E330021D16Rik*, and *GM16372*, retain DNAme in E7.5 mouse embryos (Fig. 5c–e), indicating that DNAme established in the oocyte can be maintained through gastrulation. In contrast, the CGI promoters of the orthologous human *PIWIL1* and *THEM7* genes are hypomethylated in human oocytes and blastocysts.

While many CGI promoters in human show elevated DNAme in blastocysts (Fig. 5c), deep allele-specific data are not available for this stage. However, of the 15 human meCGI promoters embedded within oocyte LITs (Supplementary Figure 4a), 4, including *ZFP90* and *SCIN* described above, were previously shown to harbor maternal-specific DNAme in placental tissues^29^. Strikingly, closer examination of the 87 placental CGI differentially methylated regions (DMRs) characterized in this study reveals that 16 are embedded within LITs (Fig. 5f), 6 of which are associated with a gene showing paternal-specific expression in human placenta^30^ (Supplementary Data 4). All 16 placental DMRs are also hypermethylated in human oocytes and show >30% methylation in blastocysts. As these DMRs are hypomethylated (<1%) in sperm, this likely reflects the persistence of DNAme on the maternal genome. Notably, the majority of these LITs initiate in LTR7 (LTR of HERVH), LTR12 (LTR of HERV9), or THE1, which are restricted to the primate lineage (Fig. 5f). In contrast, the promoters of the 16 orthologous genes are hypomethylated in mouse blastocysts (Supplementary Data 4). Taken together, these data indicate that species-specific DNAme of promoter CGIs in oocytes can be deposited as a consequence of transcription initiating in LTRs private to mouse or humans and that a subset of these CGIs resist DNAme reprogramming after fertilization in both species.

**Fig. 6:**
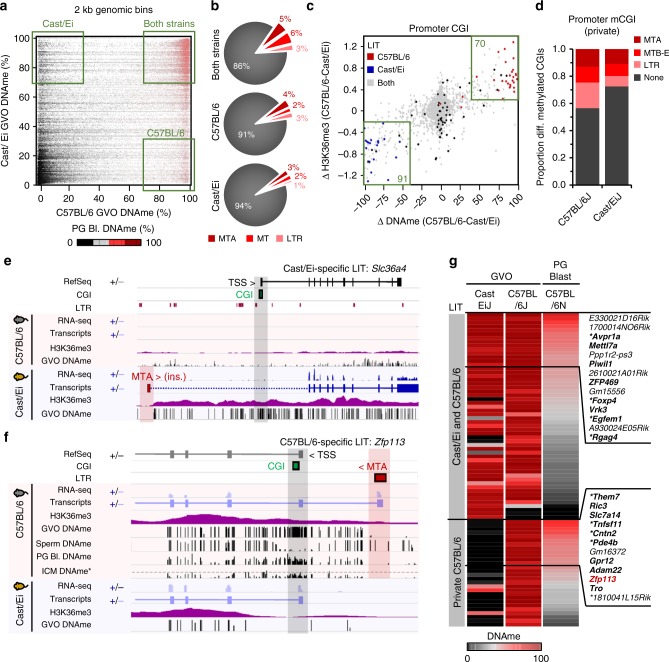
LTR polymorphisms lead to strain-specific transcripts and CGI DNAme in mouse oocytes. **a** Density plot of DNAme over 1,101,575 genome-wide 2 kb bins (>3 CpGs >1× coverage in Cast/Ei GVOs) in C57BL/6 or Cast/Ei GVOs. Regions hypermethylated (>70%) in C57BL/6 and/or Cast/Ei GVOs are highlighted. The mean percentage of DNAme in C57BL/6 parthenogenetic blastocysts (PG Bl.) is indicated as a color gradient. **b** Proportion of 2 kb bins hypermethylated in C57BL/6 and/or Cast/Ei GVOs overlapping with an LIT driven by an MTA element, another subfamily of MT element (MTB, C, D, or E) or another type of LTR. **c** Scatter plot of differential DNAme (Δ DNAme) and H3K36me3 enrichment (Δ H3K36me3) over 11,030 promoter CGIs in C57BL/6 and Cast/Ei GVOs. Differentially methylated CGIs are highlighted in green boxes. **d** Bar chart depicting each type of transcript overlapping differentially methylated promoter CGIs identified in **c**. MTA, MT, or LTR-initiated transcripts were identified by LIONs and/or manual inspection. None: no transcript or non-LTR initiated transcript. **e** Screenshot of the *Slc36a4* CGI promoter. The *Slc36a4* transcript initiates in a polymorphic MTA element (insertion in the Cast/Ei genome) in Cast/Ei GVOs. **f** Screenshot of the *Zfp113* CGI promoter. The *Zfp113* transcript initiates in an upstream MTA element in C57BL/6 GVOs but from the canonical TSS in Cast/Ei GVOs. **g** Heat map of all hypermethylated promoter CGIs (69) embedded within an LIT in C57BL/6 GVOs. DNAme levels in Cast/Ei GVOs, C57BL/6 GVOs, and C57BL/6 PG blastocysts is shown (columns), and CGIs embedded within LITs that are present in both strains or private to C57BL/6 oocytes are clustered (rows). Genes with CGI promoters retaining >45% DNAme in PG blastocysts are labeled. Bold: genes previously identified as TET targets at the implantation stage (see Fig. 5e). C57BL/6 GVO WGBS and ICM datasets from refs. ^16,26^

### Recent LTR insertions promote strain-specific DNAme

Having shown that species-specific LITs likely promote species-specific de novo DNAme of CGIs in mouse and rat, which diverged over 20 million years ago, we next addressed whether the same phenomenon may drive intra-species divergence in meCGIs. The reference mouse strain C57BL/6 (*Mus musculus domesticus*) and Southeast Asian strain Cast/Ei (*Mus musculus castaneus*), which diverged ~0.5 million years ago, harbor >20 million SNPs and >3 million indels, nearly 12,000 of which are ERVs or solo LTRs^19,31^. To dissect the potential effects of LTR-ERV polymorphisms on transcription and associated DNAme in the oocytes of these distantly related subspecies, we first generated a low-resolution PBAT library from Cast/Ei GVOs. Comparison of average DNAme levels over genome-wide 2 kb bins reveals that 3.7% and 2.5% of the genome is methylated exclusively in C57BL/6 and Cast/Ei, respectively, while 21.2% is hypermethylated in both strains (Fig. 6a). Similar to our inter-species comparisons, a greater fraction of intergenic regions shows strain-specific DNAme than intragenic regions, in accordance with the few annotated genes showing biased expression between C57BL/6 and Cast/Ei oocytes (Supplementary Figure 6a, b). To generate further evidence that these strain-specific hypermethylated regions likely gain DNAme as a result of strain-specific transcription, we generated H3K36me3 ULI-NChIP-seq data for Cast/Ei oocytes. As anticipated, regions showing divergent DNAme between Cast/Ei and C57BL/6 strains show a concomitant bias in H3K36me3 in both genic and intergenic regions (Supplementary Figure 6c).

To estimate the influence of LTR transcription on strain-specific oocyte DNAme, we identified hypermethylated regions that overlap with a predicted LIT in C57BL/6 and/or Cast/Ei oocytes (Fig. 6b). Analysis of transcript levels over the first 500 bp of all LITs identified in one or both strains reveals that many show the predicted strain-specific bias, while others appear to be expressed in both strains (Supplementary Data 1 and Supplementary Figure 6d), likely reflecting a failure to capture all de novo transcripts. Similarly, analysis of the average DNAme over LITs (>5 kb) indicates that LITs identified in only a single strain can be hypermethylated in both strains (Supplementary Figure 6d, right panel). Nevertheless, many strain-specific LITs do show a clear bias in both transcription and DNAme, indicating that such strain-specific transcription units likely promote divergent DNAme between mouse strains.

Strikingly, we identified 196 and 180 CGIs in C57BL/6 and Cast/Ei, respectively, which show strain-specific hypermethylation (Δ DNAme >40%) and H3K36me3 enrichment (Supplementary Figure 6e). While many of these differentially methylated CGIs are intergenic, 70 C57BL/6- and 91 Cast/Ei-specific meCGIs overlap with an annotated TSS (Fig. 6c). Manual inspection of this cohort of CGI promoters reveals that 20 C57BL/6-specific and 25 Cast/Ei-specific meCGI promoters likely gain DNAme as a result of transcription initiating in an LTR, often an annotated MTA or other MT subfamily (Fig. 6d). Transcription of the *Slc36a4* gene in Cast/Ei oocytes, for example, initiates in an upstream MTA element that is absent from the C57BL/6 genome, resulting in a Cast/Ei-specific hypermethylated domain overlapping the CGI promoter (Fig. 6e). Alternatively, while an MTA element upstream of the *Zfp113* CGI promoter is transcribed in both strains, it acts as an alternative TSS only in C57BL/6 oocytes (Supplementary Data 1), leading to strain-specific CGI DNAme. Notably, DNAme at this CGI is retained in C57BL/6 PG blastocysts and in F1 ICM^26^ (Fig. 6f) but is erased by E7.5 (Fig. 5e).

Approximately 40% of all meCGI promoters in C57BL/6 oocytes, including those which are hypomethylated in Cast/Ei, retain relatively high levels of DNAme (>45%) in C57BL/6 PG blastocysts (Supplementary Figure 6f). Remarkably, of these 88 persistently hypermethylated CGI promoters, 26 overlap with an LIT, 12 of which are private to C57BL/6 oocytes (Fig. 6g). Analysis of an independent WGBS dataset generated from C57BL/6×DBA ICM cells^26^ confirmed the persistence of DNAme on the maternal genome over a subset of these CGIs (Supplementary Figure 6g). Thus, similar to our observations of CGI hypermethylation associated with species-specific LITs, LTR elements can drive strain-specific differences in DNAme in oocytes, including over genic CGI promoters. Furthermore, methylation at a subset of these meCGIs is clearly retained on the maternal allele following reprogramming in the early embryo, indicating that polymorphic LTR insertions can promote heritable variation in CGI methylation even over a relatively short evolutionary timescale.

## Discussion

Previous work has revealed that LTRs acting as tissue-specific TSSs are highly abundant in various mouse and human tissues, highlighting the important role of LTR retrotransposons as sources for regulatory variation in mammals^3^. Here we ascertained the extent to which LTR-initiated transcripts impact the methylome in mouse, rat, and human oocytes. Such transcripts are likely responsible for transcription-coupled deposition of from ~11 to ~18% of total DNAme, depending on the species. The highest contribution of LITs to de novo DNAme occurs in mouse oocytes, where 40% of all LITs initiate in MTA elements, which are restricted to the mouse genome and active exclusively in the female germline. We are likely underestimating the contribution of LITs to de novo DNAme in all species evaluated due to the difficulty of identifying short first exons and/or low-level transcripts. Nevertheless, DNAme in syntenic regions is more likely to be divergent across species in regions embedded within LITs in at least one species, suggesting a role for species-specific LTR insertions in the diversification of the mammalian oocyte methylome. This phenomenon is distinct from the well-characterized A^vy^ mouse allele, where an IAP LTR acts as an alternative promoter of the *Agouti* gene when hypomethylated and shows variable inheritance of DNAme on the maternal genome^32^. A large number of LITs are also readily detected in human oocytes, including many initiating in LTR12C or LTR7 repeats, which are primate-specific and have previously been reported to act as alternative promoters in normal^33^ and cancer cells^34^.

In mouse, rat, and human oocytes, scores of hypermethylated CGIs were identified, many unique to a single species. Remarkably, many of these species-specific hypermethylated CGIs are embedded within LITs, suggesting a role for LTR-initiated transcription in the establishment of DNAme over regulatory regions. The persistence of such LIT-associated DNAme on the maternal genome in the early mouse embryo, including at CGI promoters, is readily detectable in PG blastocysts as well as F1 ICM cells. Two of the LIT-associated meCGIs identified in the mouse (see Supplementary Data 1), one intragenic to the *Cdh15* gene (embedded within an MTD-initiated chimeric transcript) and the other overlapping with the *AK008011* pseudo-gene (embedded with an RMER19B-initiated transcript), were recently identified as imprinted gametic DMRs, with methylation persisting on the maternal allele in blastocysts as well as in adult tissues^35^. Notably, the *Cdh15* gene is expressed from the paternal allele in neonatal brain and adult hypothalamus, revealing that such DNAme can impact expression from the maternal allele in somatic tissues^35^. Furthermore, DNAme of an alternative promoter of the Polycomb gene *Scml2*, which is embedded within an MTD-initiated LIT and in turn methylated in mouse oocytes, was recently shown to play a critical role in silencing of *Scml2* expression in trophoblast stem cells and early trophoblast precursors specific to placental lineages^36^. Similarly, as mentioned above, 6/16 CGIs embedded within human-specific LITs in oocytes, including *ZFP90* and *SCIN*, show persistence of maternal-specific DNAme in human placenta^29^ and are expressed exclusively from the paternal allele in this tissue^30^ (see Supplementary Data 4). These placental DMRs, however, are hypomethylated in adult tissues, likely reflecting demethylation in post-implantation embryos^30^. Taken together, these observations indicate that, in rodents and primates, lineage-specific DNAme of CGIs established as a consequence of LTR-initiated transcription in the oocyte can persist following fertilization and in turn suppress transcription from the maternal allele in adult or extraembryonic tissues. These phenomena are reminiscent of secondary epimutations but presumably impact all individuals within a species where the relevant LTR element has reached fixation.

A diverse array of LTR retrotransposon families have colonized mammalian taxa over evolutionary time^37^, with many still active in the rodent lineage. Such elements are particularly active in the female germline^6^. Thus novel oocyte-specific LITs initiating from new retrotransposon insertions may explain a significant fraction of the species-specific DNAme observed in intergenic regions. Conversely, base substitutions over time can impact transcription factor-binding sites of a specific LTR, abolishing the generation of an LIT and in turn, associated downstream DNAme. Strong evidence of the impact of recent LTR insertions on the oocyte methylome emerges when comparing the transcriptome of the reference mouse strain C57BL/6 with the wild-derived strain Cast/Ei, which yields hundreds of strain-specific LITs, nearly half emanating from MT elements. Several of these LITs overlap with CGIs, which are usually hypermethylated and enriched for H3K36me3 only in the LIT-expressing strain.

While over 12,000 LTRs are polymorphic between *M. m. domesticus* and *M. m. castaneus*^19,31^, only 875 unique LTRs insertions were identified between human and chimpanzees^38^. The primate genome has experienced a dramatic decline in ERV/LTR integration events over the past 10 million years^39^, likely explaining why the contribution of LITs to inter- and intra-species divergence in the oocyte methylome is more prevalent in rodents. Regardless, our observations reveal that LTR retrotransposons likely play an important role in shaping the methylome in oocytes of both rodent and primate lineages, including at CGI promoters. At a subset of genes, such DNAme persists beyond the blastocyst stage in the embryo proper in mouse, or in extraembryonic tissues in human, in association with transcriptional repression of the maternal allele. Though we note that retrotransposons can be upregulated in tumorigenesis, and hypermethylation of CGI promoters is a hallmark of cancer, whether LIT-associated DNAme of CGIs plays a role in human disease remains to be determined.

## Methods

### Ethical approval for animal work

Animal experimentation was in accordance with the guidelines from the Canadian Council on Animal Care (CCAC) under approval of a University of British Columbia animal care license or guidelines of the Science Council of Japan, under approval of the Institutional Animal Care and Use Committee of the Tokyo University of Agriculture. No randomization was used in this study. The investigators were not blinded during animal experiments.

### Oocyte isolation

Mouse GVOs were isolated from 5-to-10-week-old C57BL/6J or Cast/EiJ females following mechanical dissociation of the ovaries. Rat GVOs were isolated from 8-week-old Sprague Dawley females following mechanical dissociation of the ovaries. Fully grown GVOs were selected based on their size (>80 μm) and nuclear morphology. Rat metaphase-II (MII) oocytes were isolated from the ovarian follicles of 10-week-old Wistar Han females (BrlHan:WIST@Jcl, Clea Japan).

### Sperm isolation

Rat sperm was released from the cauda epididymis of 10-week-old Wistar Han males. Following chromatin decondensation with dithiothreitol, DNA was purified by phenol–chloroform extraction followed by ethanol precipitation.

### Blastocyst isolation

Both mouse normal and PG blastocysts were obtained from C57BL/6N mice (Clea Japan). Normal zygotes were isolated by in vitro fertilization of ovulated oocytes. PG zygotes were constructed by stimulating cumulus-free oocytes with strontium chloride solution, which contains cytochalasin B to prevent extrusion of the second polar body. Normal and PG zygotes were cultured to the blastocyst stage in sperm-free KSOM medium (Merck Millipore) for 3 and 4 days, respectively. Each blastocyst was evaluated for expansion, ICM, and trophectoderm appearance to select non-arrested blastocysts.

### Post-bisulfite adaptor tagging

Twenty C57BL/6N blastocysts, 20 C57BL/6N PG blastocysts, 800 Wistar Han oocytes, or 372 CAST/EiJ GVOs were spiked with 0.03 ng of unmethylated lambda phage DNA, an external control for monitoring bisulfite conversion rate, placed in a lysis solution (0.1% sodium dodecyl sulfate (SDS), 1 mg/mL proteinase K) for 60 min at 37 °C and then 15 min at 98 °C. The purified 100 ng of Wistar Han sperm were also spiked with 0.5 ng of unmethylated lambda phage DNA. Bisulfite conversion was performed using the EZ DNA Methylation-Gold Kit (Zymo Research), and amplification-free WGBS libraries were constructed using the PBAT method^11,15^ (also available from http://crest-ihec.jp/english/epigenome/index.html). Briefly, bisulfite-treated DNA was re-annealed to double-stranded DNA using Klenow fragments (3′–5′ exo−; New England Biolabs) with the modified Bio-PEA2-N4 primer: 5′-biotin-ACA CTC TTT CCC TAC ACG ACG CTC TTC CGA TCT NNN N-3′ (N = A, C, G, or T) for mouse samples and rat sperm, or the Bio-PEA2-W4N4 primer: 5′-biotin-ACA CTC TTT CCC TAC ACG ACG CTC TTC CGA TCT WWW WNN NN-3′ (W = A or T) for rat oocytes. The synthesized first strands were captured using Dynabeads M280 Streptavidin (Thermo Fisher Scientific) and re-annealed to double-stranded DNA again using Klenow fragments (3′–5′ exo−) with PE-reverse-N4 primer: 5′-CAA GCA GAA GAC GGC ATA CGA GAT NNN N-3′ for mouse samples, or PE-index-N4 or PE-index-W4N4 primers: 5′-CAA GCA GAA GAC GGC ATA CGA GAT XXX XXX GTA AAA CGA CGG CCA GCA GGA AAC AGC TAT GAC N4 or W4N4‐3′ (in which XXX XXX stands for the index sequence of each primer) for rat sperm and oocytes, respectively. Finally, template DNA strands were synthesized as cDNA with the second strand using Phusion Hot Start High-Fidelity DNA Polymerase II (New England Biolabs) with the Illumina primer PE 1.0 (5′-AAT GAT ACG GCG ACC ACC GAG ATC TAC ACT CTT TCC CTA CAC GAC GCT CTT CCG ATC T-3′). The concentrations of PBAT libraries were determined by quantitative PCR using the KAPA Library Quantification Kit for Illumina platforms (Kapa Biosystems). The PhiX v2 Control Kit (Illumina) was used as a standard for quantification. The constructed PBAT libraries were subject to massively parallel sequencing on an Illumina HiSeq 2500 platform to generate 100-nt single-end (mouse datasets) or paired-end (rat datasets) sequence reads. Before alignment, each random sequence (N4 or W4N4) are trimmed from the sequence data sets. Our PBAT data were aligned to each genome references using Bismark^40^. The bisulfite conversion rate as determined by analysis of lambda DNA was over 99%. Biological replicates were combined to increase resolution and coverage. Methylated domain landscape plots were generated using changepoint detection analysis^41^. For all PBAT and WGBS datasets (except Cast/Ei GVO PBAT), we calculated average DNAme over a given set of genomic coordinates with >4 CpGs with >5× coverage. For our low-resolution Cast/Ei GVO PBAT datasets, we calculated average DNAme over a given set of genomic coordinates with >3 CpGs with >1× coverage. Allele-specific alignment of published WGBS data generated from C57BL/6×DBA ICM cells was performed using our allele-specific pipeline MEA^42^.

### Total RNA-sequencing and transcriptome analysis

Total RNA was isolated from 100 to 200 mouse or rat oocytes using TriReagent, and ribosomal RNA was depleted using the NEB Next rRNA Depletion Kit according to the manufacturer’s instruction. Double-stranded cDNA was synthesized using NEB Next first-strand and second-strand synthesis modules. Libraries were constructed using a custom protocol^18^. Following end-repair and A-tailing, universal Illumina adapters were ligated and amplified for 10–12 PCR cycles using primer 1.0 (5′- AAT GAT ACG GCG ACC ACC GAG ATC TAC ACT CTT TCC CTA CAC GAC GCT CTT CCG ATC T -3′) and indexed primer 2.0 (5′-CAAGCAGAAGACGGCATACGAGATCXXXXXXGGTCTCGGCATTCCTGCTGAACCGCTCTTCCGATCT-3′, where XXXXXX represents the index). In all, 75 or 100 bp paired-end libraries were sequenced on the NextSeq 500 or HiSeq 2500 platform according to the manufacturer’s instructions. Paired-end reads were trimmed using Trimmomatic^43^ v.0.32 and aligned to the mm10 (mouse), hg19 (human), and rn6 (rat) assemblies using STAR v.2.4.0.i^44^. Library technical quality was assessed using Picard-tools (http://broadinstitute.github.io/picard) v.1.92 and Samtools (http://www.htslib.org/) v.1.1. PCR duplicates were filtered out. Sequence alignment maps were converted to bedGraph and wiggle formats using Bedtools^45^ v2.22.1 and UCSC binary bedGraphToBigWig. Normalized read counts was produced using VisRseq^46^ v.0.9.15 over Ensembl gene or Repeat Masker annotations of each species. Reproducibility was evaluated by comparison of canonical gene transcription levels and were combined for subsequent analyses. For comparison of gene expression levels across species, canonical gene expression levels were performed over merged isoforms of all annotated Ensembl genes in each individual species using the SeqMonk RNA-seq analysis pipeline, and correlation analysis over 11,186 annotated syntenic Ensembl genes (BioMart) was performed using Morpheus (https://software.broadinstitute.org/morpheus/). For cross-species comparisons (species A vs species B) over Ensembl gene orthologs, *Z*-scores were calculated as [(FPKM A − FPKM B)/(SQRT(FPKM A + FPKM B) + 0.01)]. De novo transcriptome assemblies from strand-specific RNA-seq libraries were produced using Cufflinks v.2.1.1^21^ with default parameters. The contribution of endogenous retrovirus initiated transcripts was assessed using LIONS^20^. Briefly, 5’ ends of de novo assembled transcripts (which include canonical and chimeric transcripts) were classified based on overlaps with UCSC RepeatMasker and Ensembl gene annotations. For transcripts with 5’ LTR-driven promoters, the contribution of LTR-driven transcription was assessed by calculating read coverage on exon1 (the LTR) relative to the first annotated canonical exon. To obtain the list of LITs with high specificity, only transcripts with the Up or UpEdge LTR (per LIONS raw output) were taken into consideration. For manual inspection of LITs over specific CGI promoters, LITs were either identified by manually validating transcripts with EInside LTR contribution in the LIONS raw output or by intersecting de novo transcripts with the boundaries of hypermethylated domains.

### Chromatin immunoprecipitation (ChIP)-sequencing

Following GVO isolation, the Zona Pellucida was dissolved by 4–5 passages through acid Tyrode’s solution and oocytes were neutralized in M2 media. Oocytes were then transferred to nuclear isolation buffer (Sigma) and flash frozen in liquid nitrogen. H3K36me3 ChIP-seq libraries were prepared from ~200 GVOs using ULI-NChIP-seq^18^. Briefly, following MNase digestion (NEB), chromatin was diluted in native ChIP buffer and incubated with 0.15 μg of anti-H3K36me3 (Abcam 9050) and 5 μl of protein A: protein G 1:1 Dynabeads (Thermo Fisher). Following elution in 0.1 nM NaCO_3_ and 1% SDS, DNA was extracted using phenol:chloroform and precipitated in 75% ethanol, followed by library construction as described above. Libraries were sequenced (75 bp paired-end) on a NextSeq 500 according to the manufacturer’s protocols. Reads were trimmed using Trimmomatic^43^ v.0.32 and aligned to the mm10 (mouse), hg19 (human), and rn6 (rat) assemblies using Bowtie2^47^ v.2.2.3 with soft-clipping enabled. Library technical quality was assessed using Picard-tools (http://broadinstitute.github.io/picard) v.1.92 and Samtools (http://www.htslib.org/) v.1.1. PCR duplicates and alignments with mapping quality <10 were filtered out. Sequence alignment maps were converted to bedGraph and wiggle formats using Bedtools^45^ v2.22.1 and UCSC binary bedGraphToBigWig. Normalized read counts was produced using VisRseq v.0.9.15^46^.

### Intra- and inter-species annotations

Gene annotations for mouse (mm10), rat (rn6), and human (hg19) were obtained from BioMart-Ensembl, and syntenic gene annotation was generated using the ortholog function. All transcript isoforms were merged. Syntenic genomic bins in the mouse, rat, and human genomes were generated using the reciprocal best method using mouse–rat, mouse–human, rat–mouse, and human–mouse chains obtained from UCSC. Only genomic bins present in all three species and with sufficient PBAT/WGBS coverage (see below) were used for inter-species comparison. Genic syntenic genomic bins were defined as regions overlapping with an annotated Ensembl gene (TSS to TTS + 2 kb) in all three species; intergenic syntenic genomic bins were defined as regions with no Ensembl gene overlap in all three species. CGI annotations for mouse (mm10), rat (rn6), and human (hg19) were obtained from UCSC Table Browser and intersected with Ensembl Gene annotations for each cognate species. Promoter CGIs were defined as CGIs overlapping an annotated Ensembl TSS (TSS ± 100 bp), and intragenic CGIs were defined as overlapping an annotated Ensembl. For syntenic CGI annotation, genomic coordinates for rat or human CGIs were converted to mm10 coordinates using the UCSC LiftOver tool and compared to the mm10 CGI annotation. CGIs with >0.5 identity (see Fig. 4b) were defined as syntenic.

### Data visualization

Density and scatter plots were generated using VisRSeq^46^ v.0.9.15, heat maps were generated using ChASe^48,49^ v.1.0.11, and Venn diagrams were generated using BioVenn^50^. Genome browser screenshots were generated using Integrated Genome Viewer^51^, and bar graphs and pie charts were generated using Microsoft Excel.

### Data availability

Details of the datasets generated are presented in Supplementary Table 3. ChIP-seq and RNA-seq datasets have been deposited at the Gene Expression Omnibus database (accession GSE112622) and WGBS datasets have been deposited at the DNA Databank of Japan (accession nos. DRA006642, DRA006679, and DRA006680).

## Electronic supplementary material

Supplementary Information

Peer Review File

Description of Additional Supplementary Files

Supplementary Data 1

Supplementary Data 2

Supplementary Data 3

Supplementary Data 4
