# Supplementary material for: LTR retrotransposons transcribed in oocytes drive species-specific and heritable changes in DNA methylation
Source: Nat Commun. 2018 Aug 20;9:3331. doi: 10.1038/s41467-018-05841-x (PMC6102241; doi:10.1038/s41467-018-05841-x)
Supplement: Supplementary file 1 — Supplementary Information [file 41467_2018_5841_MOESM1_ESM.pdf]

SUPPLEMENTARY INFORMATION

LTR retrotransposons transcribed in oocytes drive species-specific, heritable changes in DNA methylation

Julie Brind'Amour[1], Hisato Kobayashi[2*], Julien Richard Albert[1], Kenjiro Shirane[1], Akihiko Sakashita[3,4], Asuka Kamio[2,5], Aaron Bogutz[1], Tasuku Koike[3], Mohammad M. Karimi[1,6], Louis Lefebvre[1], Tomohiro Kono[3] and Matthew C. Lorincz[1*]

Included:

Supplementary Tables 1-3

Supplementary Figures 1-6

Supplementary References

	Genic	Intergenic	% genome covered (no Y chr)	% genic bins	% hypermethylated bins overlapping an annotated gene
**Mouse**	934,621	1,132,257	78.48	45.22	68.56
**Rat**	794,069	1,195,429	75.12	39.91	64.36
**Human**	1,024,005	1,132,257	70.98	47.49	61.94

Supplementary Table 1: Genomic distribution of all genomic 1kb bins with sufficient WGBS coverage oocytes

	Genic	Intergenic	% genome covered (no Y chr)	% genic bins
**Mouse**	259,385	173,726	16.44	59.89
**Rat**	220,164	212,947	16.35	50.83
**Human**	267,761	165,350	14.26	61.82
**All species***	206,821	140,314	-	59.58

**bins consistently genic or intergenic in all 3 species*

Supplementary Table 2: Genomic distribution of syntenic 1kb bins with sufficient WGBS coverage

	Strain	Data type			Platform	Read length	Comments	Accession	Total reads/read pairs)	Non duplicate reads/read pairs)	Cov.	Ref.
		WG BS	RNA -seq	ChIP -seq								
**Mouse**												
**NGO**	C57BL/6N	■			HiSeq 2000	100 SE		DRA000570	264,687,922	175,463,324	6.42	1
**GVO**	C57BL/6N	■			HiSeq 2000	100 SE		DRA000570	486,723,254	288,189,658	10.54	1
	Cast/EiJ	■			HiSeq 2500	100 SE	low coverage	DRA006680	144,701,932	31,396,730	0.85	This manuscript
	C57BL/6J		■		NextSeq 500	76 PE	2x replicates	GSE112622	119,180,710	113,908,721	nr	This manuscript
	Cast/EiJ		■		NextSeq 500	76 PE	2x replicates	GSE112622	93,367,582	87,545,451	nr	This manuscript
	C57BL/6J			■	NextSeq 500	76 PE	H3K36me3	GSE112622	58,349,727	42,095,016	nr	This manuscript
	C57BL/6N			■	HiSeq 2500	101 PE	H3K4me3	GSE71434	33,131,285	19,455,229	nr	2
	Cast/EiJ			■	NextSeq 500	76 PE	H3K36me3	GSE112622	66,829,852	46,352,441	nr	This manuscript
**Sperm**	C57BL/6N	■			HiSeq 2000	100 SE		DRA000484	871,441,800	542,186,944	19.8	3
**ICM**	C57BL/6N x DBA2J	■			HiSeq 2000			GSE56697	-	-	-	4
**Blast.**	C57BL/6N	■			HiSeq 2500	100 SE	2x replicates	DRA006679	808,948,430	510,712,920	18.7	This manuscript
**PG Blast.**	C57BL/6N	■			HiSeq 2500	100 SE	2x replicates	DRA006679	500,093,650	349,567,290	12.8	This manuscript
**Rat**												
**GVO**	SD		■		NextSeq 500	76 PE	2x replicates	GSE112622	117,611,250	113,029,636	nr	This manuscript
**MII**	WH		■		HiSeq 2500	100 PE	2x replicates	GSE112622	178,218,899	136,975,711	nr	This manuscript
	WH	■			HiSeq 2500	100 PE	4x replicates	DRA006642	804,149,462	244,949,332	9.1	This manuscript
**Sperm**	WH	■			HiSeq 2500	100 PE	3x replicates	DRA006642	882,273,380	288,991,198	10.6	This manuscript
**Human**												
**10 wk F. PGC**	-	■			HiSeq 2500	100 PE		GSE63818	537,555,866	180,528,778	-	5
**GVO**	-		■		HiSeq 2000	100 PE		GSE85632	-	-	-	6
**MII**	-		■		HiSeq 2000	100 PE		GSE85632	-	-	-	6
**GVO + MI**	-	■			HiSeq 2000/ 2500	100 SE		DRA003802	-	224,236,188	7.0	7
**Sperm**		■			HiSeq 2000/ 2500	100 SE		DRA003802	-	782,367,257	24.5	7
**Blast.**	-	■			HiSeq 2000/ 2500	100 SE		DRA003802	-	750,044,631	23.5	7

Supplementary Table 3: Datasets summary
PG: parthenogenetic; Blast: blastocyst; Cov.: coverage

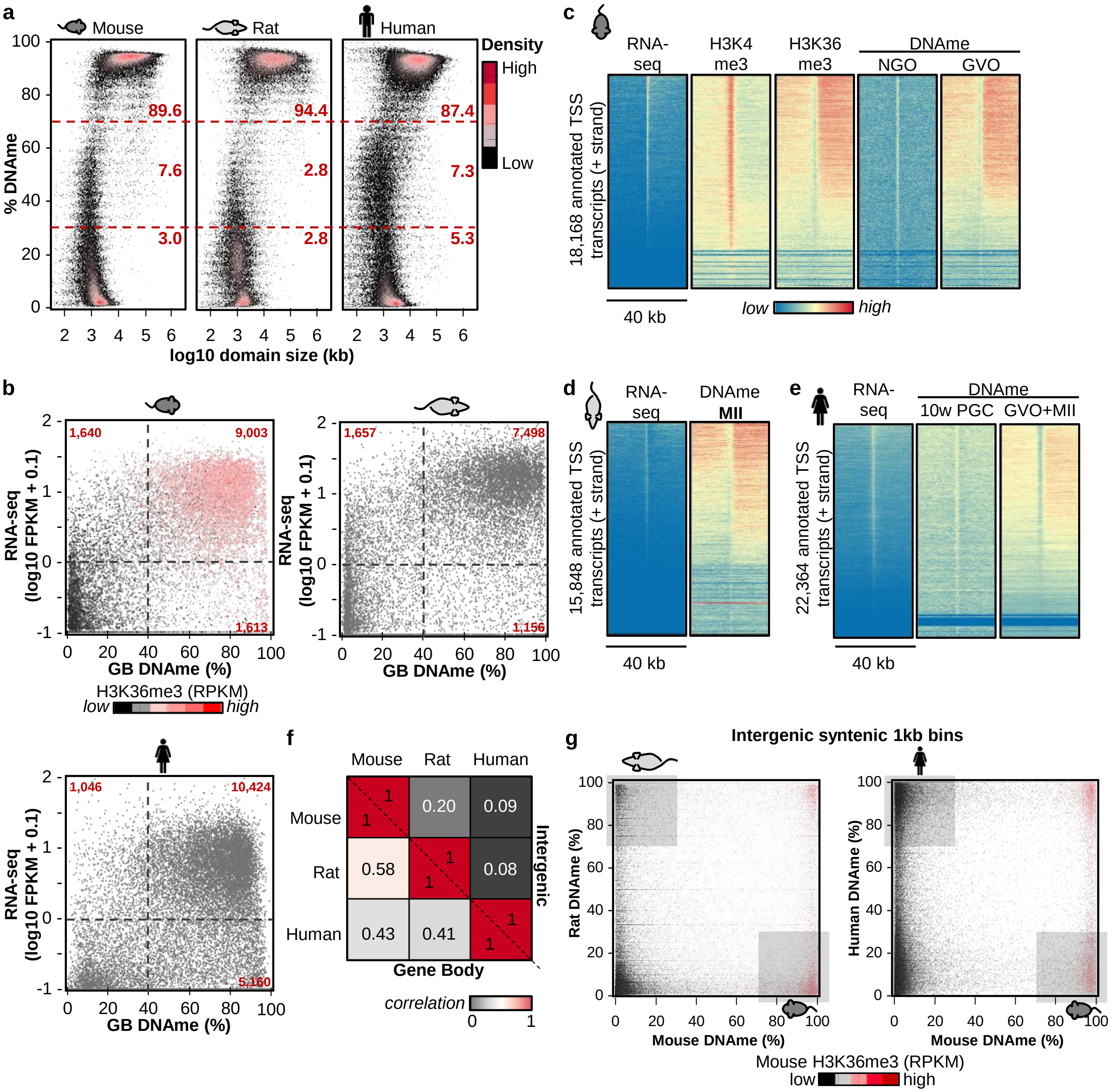

Supplementary Figure 1. Correlation between DNAme and transcription in mammalian oocytes

Supplementary Figure 1. a. Density plots depicting the distribution of DNAme in mouse, rat or human sperm. The proportion of the genome with low (<30%), intermediate (30-70%) or high (>70%) levels of DNAme for each species is indicated in red. **b.** Comparison between gene transcription and gene body (GB) DNAme in mouse (left), rat (right) and human (bottom) oocytes. H3K36me3 enrichment over GBs in mouse oocytes is illustrated as a color gradient. 18,023 (mouse), 16,367 (rat) and 22,822 (human) Ensembl genes (> 5kb) with > 5 CpGs covered are illustrated, and the number of genes transcribed (>1 FPKM) and/or DNA methylated (> 40% DNAme) are indicated in red. **c.** Heatmaps depicting the correlation between transcription and downstream DNAme in mouse oocytes at 18,168 annotated TSS sorted by gene transcription level. H3K4me3 enrichment overlaps with the TSS, whereas H3K36me3 and *de novo* DNAme are observed downstream of the initiation site. NGO: non-growing oocytes; GVO: germinal vesicle oocytes. **d.** Heatmaps depicting the correlation between transcription and downstream DNAme in rat oocytes at 15,848 annotated TSS sorted by gene transcription level. MII: metaphase-II oocytes. **e.** Heatmaps depicting the correlation between transcription and downstream DNAme in human 10 week old female PGCs and oocytes at 22,364 annotated TSS sorted by gene transcription level. **f.** Heatmaps depicting the correlation between DNAme patterns in mouse, rat and human oocytes over syntenic genomic regions (1kb bins) that overlap annotated genes (Gene Body) or intergenic regions (Intergenic). **g.** Scatter plot comparing DNAme over intergenic 1kb bins in mouse versus rat (left) or human (right) oocytes. 140,171 syntenic bins (>5 CpGs; >5x coverage) are included. H3K36me3 enrichment over each region in mouse oocytes is depicted as a color heat map. Private hypermethylated genomic regions for mouse, rat or human are highlighted in grey. Mouse and human WGBS datasets analyzed from refs. 3,7,1 human RNA-seq data from ref. 6 and mouse H3K4me3 from ref 2.

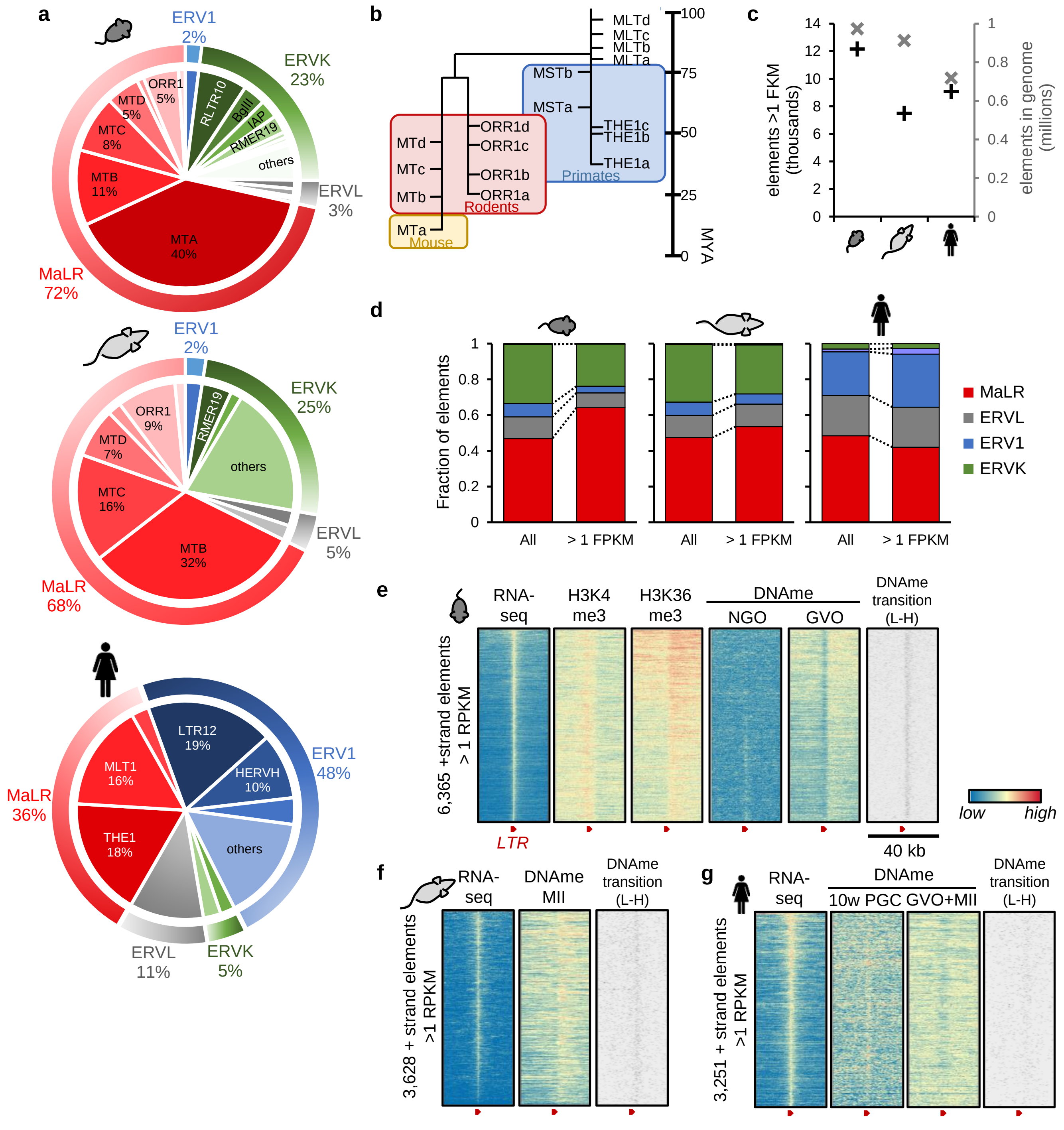

Supplementary Figure 2. LTR transcription impacts DNAme in mammalian oocytes

Supplementary Figure 2. a. Retrotransposon families driving LITs in mouse, rat, and human oocytes. **b.** Evolution of MaLR retrotransposons throughout the rodent and primate lineage[8]. Note that MT elements are absent from the primate lineage. **c.** Dot plot depicting the total number (**x**) of LTR-retrotransposons in each genome and the number of actively transcribed elements (>1 RPKM; **+**). **d.** Bar charts of the relative contribution of LTR retrotransposons of each class transcribed (> 1 FPKM) in mouse, rat and human oocytes relative to their genomic abundance in each respective genome. **e.** Heat maps depicting the correlation between transcription and downstream DNAme in mouse oocytes flanking 6,365 forward strand LTR elements transcribed >1 RPKM in mouse oocytes. H3K4me3 enrichment overlaps with the LTR. H3K36me3, DNAme and the 5' edge of hypermethylated domains (DNAme transition L-H), identified in Fig. 1B, are observed downstream of the LTR. **f.** Heat maps depicting DNAme enrichment around 3,628 forward strand LTR elements transcribed (>1 RPKM) in rat oocytes. DNAme and the 5' edge of hypermethylated domains (DNAme transition L-H) identified in Fig. 1B are observed downstream of the active LTR. **g.** Heat maps depicting DNAme enrichment in human female PGCs isolated at 10-weeks and mature oocytes (GVOs + MII) around 3,251 forward strand LTR elements transcribed (>1 RPKM) in human oocytes. Little DNAme and few hypermethylated domains (DNAme transition L-H) are observed downstream of transcribed solo LTRs. Mouse and human WGBS datasets analyzed from refs. 3,7,1, human RNA-seq data from ref. 6 and mouse H3K4me3 from ref 2.

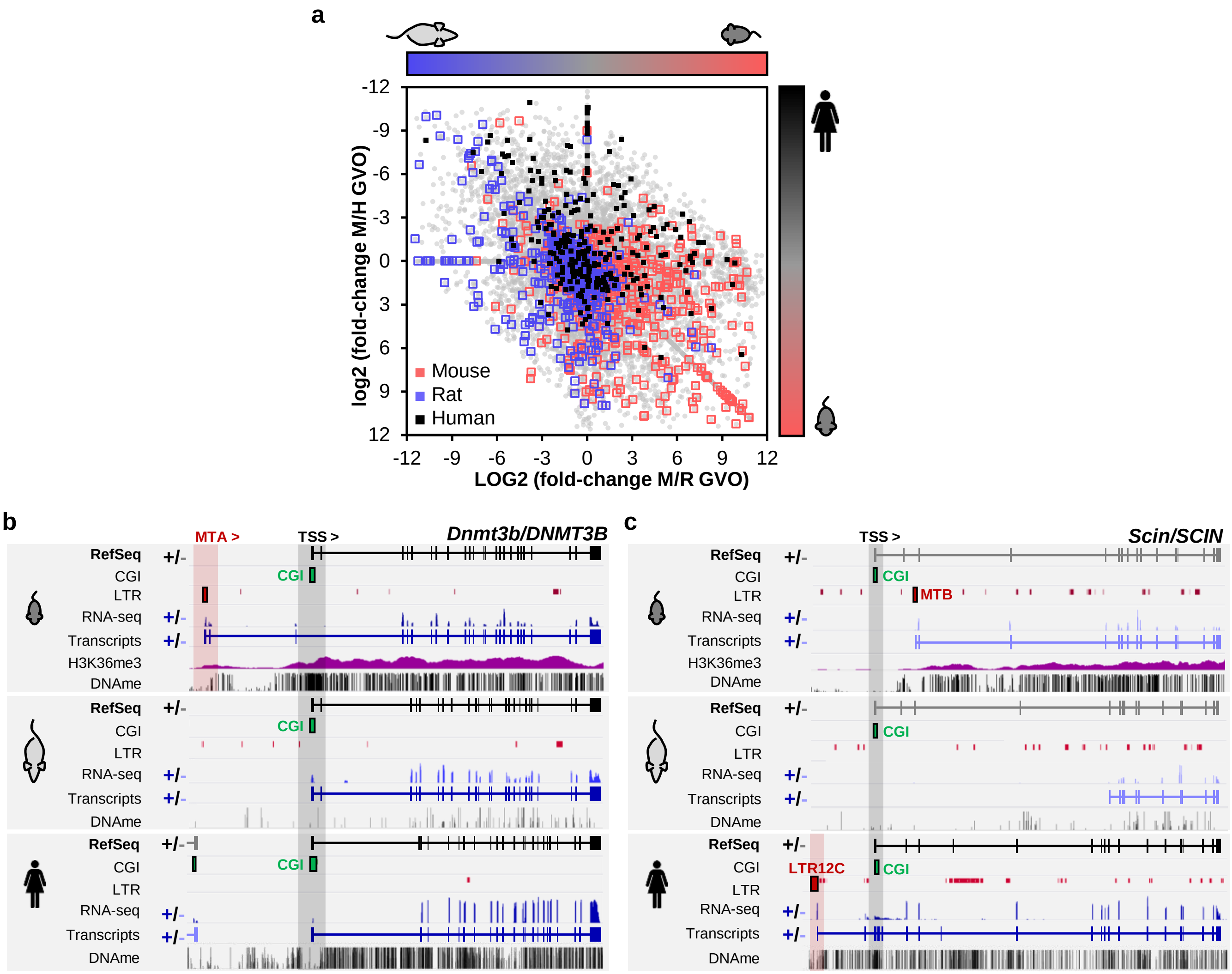

Supplementary Figure 3. LTR contribution to chimaeric transcripts in oocytes. **a.** Comparison between gene transcription in mouse, rat and human GVOs (grey). Highlighted are genes with an LIT identified in mouse (red), rat (blue) or human (black). **b.** Genome browser screenshots of syntenic *Dnmt3b/DNMT3B* loci, including transcription and DNAme in mouse, rat and human oocytes. A mouse transcript initiates in an upstream MTA element, while rat and human transcripts initiate at the canonical TSS. **c**. Genome browser screenshots of syntenic *scin/SCIN* loci, showing transcription initiation from an RLTR12c element unique to the human genome leading to *de novo* DNAme of the canonical *SCIN* CGI promoter. Mouse and human WGBS datasets analyzed from refs. 3,7,1 and human RNA-seq data from ref. 6.

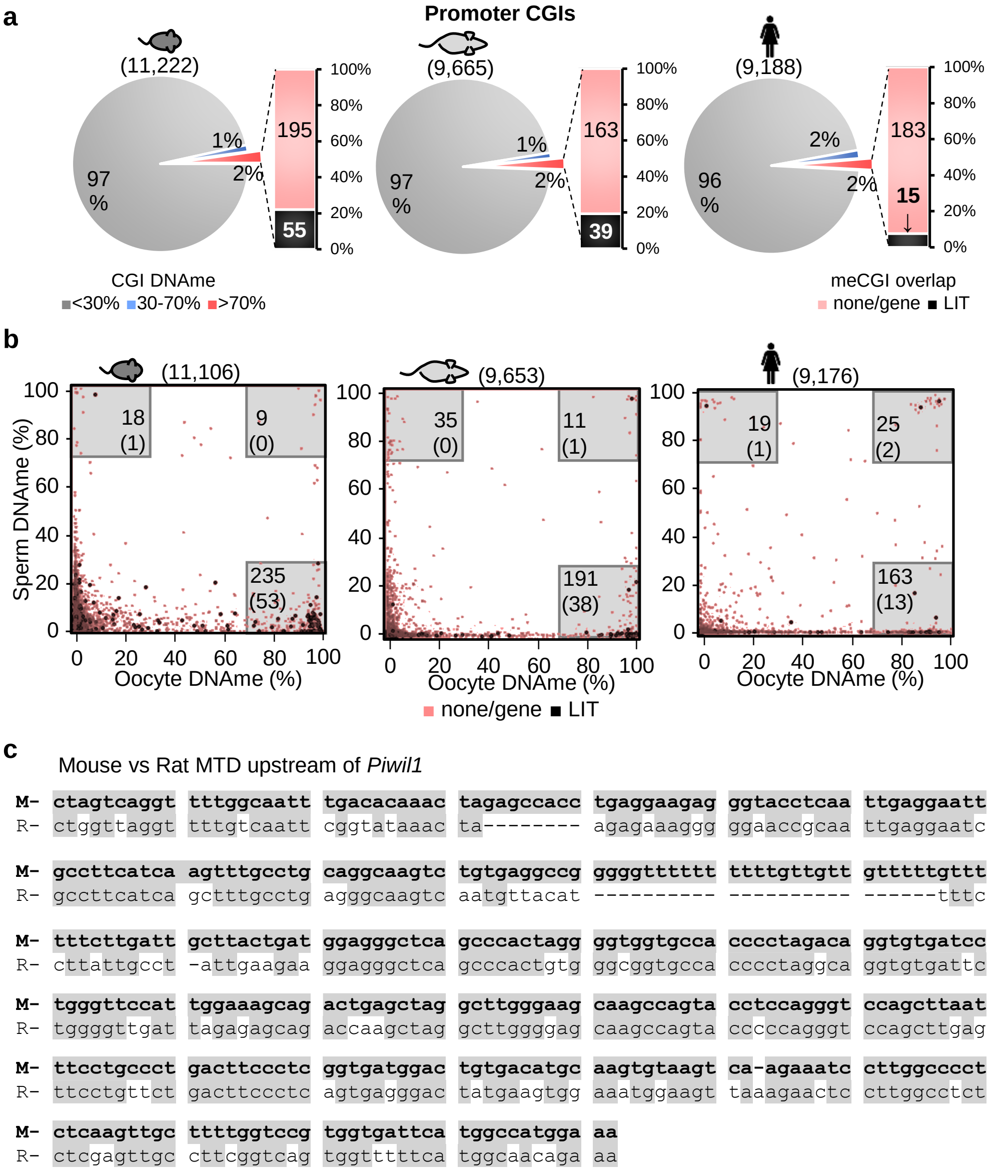

Supplementary Figure 4. LTR-initiated transcription leads to CGI methylation in oocytes. **a.** Proportion of all promoter CGIs with low (<30%), intermediate (30-70%) or high DNAme levels in mouse (left), rat (center) or human (right) oocytes. The proportion of hypermethylated CGIs (>70% DNAme) located inside an LIT is indicated on the bar chart on the right. **b.** Scatter plots of oocyte vs sperm CGI DNAme at promoter CGIs in mouse, rat and human. Promoter CGIs overlapped by an LIT in oocytes are highlighted in black. The number of oocyte- or sperm-specific hypermethylated CGIs is indicated in the grey boxes, with the number of meCGIs overlapped by an LIT indicated in brackets. **c.** Aligned sequences for the MTD element utilized as an alternative TSS for the *Piwil1* gene in mouse oocytes and putative orthologous MTD in the rat genome, which is transcriptionally silent in rat oocytes. Mouse and human WGBS datasets from refs. 3,7,1 and 4.

a

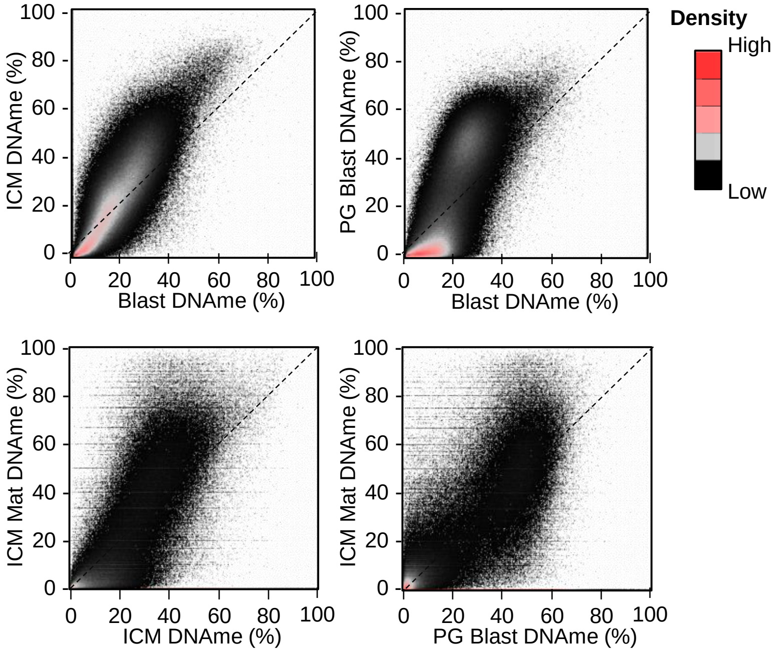

b

	Blast	PG Blast	ICM	ICM Mat
**Blast**	1	0.79	0.84	0.72
**PG Blast**	0.79	1	0.77	0.85
**ICM**	0.84	0.77	1	0.79
**ICM Mat**	0.72	0.85	0.79	1

Supplementary Figure 5. Parthenogenetic blastocysts recapitulate DNA methylation reprogramming on the maternal genome. **a.** Density plots comparing DNAme levels between blastocysts, PG blastocysts, ICM, as well as on the maternal allele of ICM over genome-wide 2kb bins. **b.** Pearson correlation coefficients between DNAme levels in blastocysts, PG blastocysts, ICM, as well as on the maternal allele of ICM over genome-wide 2kb bins.

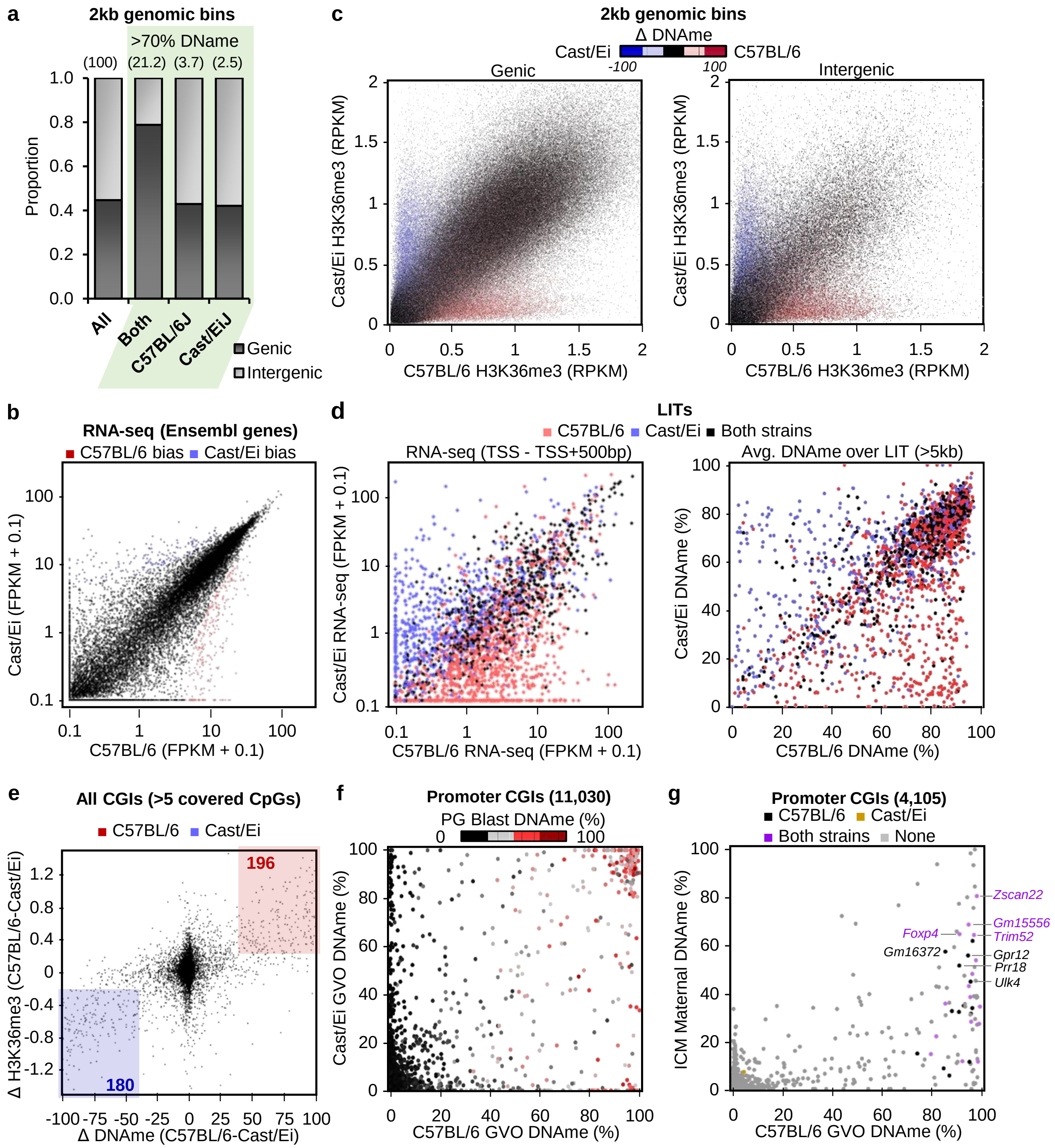

Supplementary Figure 6. Recent LTR integrations lead to strain-specific transcripts and *de novo* CGI DNAme in mouse oocytes

Supplementary Figure 6. a. Stacked bar chart depicting the proportion of hypermethylated genomic 2kb genomic bins in C57BL/6 and/or Cast/Ei oocytes that overlap an annotated Ensembl gene or that are found in intergenic regions. **b.** Pair-wise comparison of oocyte transcript levels over annotated Ensembl genes in mouse strains C57BL/6 and Cast/Ei. Genes with enriched transcription levels in each species (FPKM > 1; Z-score > 2; fold-change >2) are highlighted in red (C57BL/6) or blue (Cast/Ei). **c.** Scatter plots of H3K36me3 enrichment in C57BL/6 or Cast/Ei GVOs over genic (left) or intergenic (right) genomic 2kb bins. The differential DNAme (Δ DNAme) between C57BL/6 and Cast/Ei GVOs is illustrated as a color gradient. **d.** Scatter plots comparing C57BL/6 and Cast/Ei transcription, DNAme and H3K36me3 levels over all LITs identified with LIONs. Normalized RNA-seq signal (FPKM) was calculated over the first 500 bp of the LIT to distinguish from isoforms with downstream non-LTR transcription initiation, and average DNAme (%) was calculated over the entire length of the LIT. Red: LITs identified in C57BL/6 oocytes; blue: LITs identified in Cast/Ei oocytes; black: LITs identified in both strains. **e.** Scatter plot depicting differential DNAme (Δ DNAme) and H3K36me3 enrichment over 15,430 CGIs with >3 covered CpGs (1X coverage in the Cast/Ei GVOs). High confidence differentially methylated CGIs are highlighted in red (C57BL/6-specific) or blue (Cast/Ei specific). **f.** Scatter plot depicting C57BL/6 and Cast/Ei GVO, as well as C57BL/6 PG blastocysts (color gradient) DNAme over 11,030 promoter CGIs. A fraction of the CGIs hypermethylated in C57BL/6 GVOs, including those hypomethylated in Cast/Ei GVOs, retain DNAme after fertilization. The CGIs hypermethylated exclusively in Cast/Ei GVOs harbor low DNAme levels in C57BL/6 parthenogenic blastocysts. **g.** Scatter plot depicting C57BL/6 GVO vs maternal ICM (C57BL/6 x DBA cross) DNAme over 4,105 informative promoter CGIs. CGIs overlapped by an LIT identified in C57BL/6 and/or Cast/Ei oocytes are highlighted in purple (both strains), black (C57BL/6 private) or yellow (Cast/Ei private). C57BL/6 GVO WGBS dataset analyzed from ref. 1 and ICM from ref. 4.

SUPPLEMENTARY REFERENCES

1. Shirane, K. *et al.* Mouse oocyte methylomes at base resolution reveal genome-wide accumulation of non-CpG methylation and role of DNA methyltransferases. *PLoS Genet* **9,** e1003439 (2013).

2. Zhang, B. *et al.* Allelic reprogramming of the histone modification H3K4me3 in early mammalian development. *Nature* **537,** 553–557 (2016).

3. Kobayashi, H. *et al.* Contribution of intragenic DNA methylation in mouse gametic DNA methylomes to establish oocyte-specific heritable marks. *PLoS Genet* **8,** e1002440 (2012).

4. Wang, L. *et al.* Programming and inheritance of parental DNA methylomes in mammals. *Cell* **157,** 979–991 (2014).

5. Guo, F. *et al.* The Transcriptome and DNA Methylome Landscapes of Human Primordial Germ Cells. *Cell* **161,** 1437–1452 (2015).

6. Hendrickson, P. G. *et al.* Conserved roles of mouse DUX and human DUX4 in activating cleavage-stage genes and MERVL/HERVL retrotransposons. *Nat Genet* **49,** 925–934 (2017).

7. Okae, H. *et al.* Genome-wide analysis of DNA methylation dynamics during early human development. *PLoS Genet* **10,** e1004868 (2014).

8. Smit, A. Identification of a new, abundant superfamily of mammalian LTR-transposons. *Nucleic Acids Research* **21,** 1863–1872 (1993).

9. Hanna, C. W. *et al.* Pervasive polymorphic imprinted methylation in the human placenta. *Genome Research* **26,** 756–767 (2016).

10. Sanchez-Delgado, M. *et al.* Absence of Maternal Methylation in Biparental Hydatidiform Moles from Women with NLRP7 Maternal-Effect Mutations Reveals Widespread Placenta-Specific Imprinting. *PLoS Genet* **11,** e1005644 (2015).
