# Supplementary material for: LTR retrotransposons transcribed in oocytes drive species-specific and heritable changes in DNA methylation
Source: Nat Commun. 2018 Aug 20;9:3331. doi: 10.1038/s41467-018-05841-x (PMC6102241; doi:10.1038/s41467-018-05841-x)
Supplement: Supplementary file 3 — Description of Additional Supplementary Files [file 41467_2018_5841_MOESM3_ESM.pdf]

DESCRIPTION OF ADDITIONAL SUPPLEMENTARY FILES

File Name: Supplementary Data 1:

Description: LTR-initiated transcripts (LITs) identified in mouse oocytes. LITs were identified in mouse oocytes using the LIONs pipeline, as described in the Methods section. Genomic coordinates for the full-length transcript are included, as well as the repeat ID (start of the repeat annotation). For LITs overlapping an annotated gene, the overlap type (s: sense, as: antisense, c: combination, i: intergenic) is indicated. The strain where the LIT has been identified (Libraries identified), and in which RNA-seq reads at the LIT initiation are detected (Strain(s) transcribed), is also listed.

File Name: Supplementary Data 2:

Description: LTR-initiated transcripts (LITs) identified in rat oocytes. LITs were identified in rat oocytes using the LIONs pipeline, as described in the Methods section. Genomic coordinates for the full-length transcript are included, as well as the repeat ID (start of the repeat annotation). For LITs overlapping an annotated gene, the overlap type (s: sense, as: antisense, c: combination, i: intergenic) is indicated. The strain where the LIT has been identified (Libraries identified), and in which RNA-seq reads at the LIT initiation are detected (Strain(s) transcribed), is also listed. SD: Sprague-Dawley, WH: Wistar Han.

File Name: Supplementary Data 3:

Description: LTR-initiated transcripts (LITs) identified in human oocytes. LITs were identified in human oocytes using the LIONs pipeline, as described in the Methods section. Genomic coordinates for the full-length transcript are included, as well as the repeat ID (start of the repeat annotation). For LITs overlapping an annotated gene, the overlap type (s: sense, as: antisense, c: combination, i: intergenic) is indicated, and the dataset (MII or GVO) where the LIT was identified is also listed (Libraries identified).

File Name: Supplementary Data 4:

Description: Human gametic DMRs retaining maternal DNAme in the placenta. List of 87 previously identified (supplementary reference 9) human gametic DMRs showing DNAme retention in the placenta. DMRs overlapped by an LIT in human oocytes are highlighted, and the LTR or ERV internal region in which the LITs initiate are indicated

DESCRIPTION OF ADDITIONAL SUPPLEMENTARY FILES

(LTR at 5' end of LIT). LITs were identified using either the LIONs pipeline (LIONS) or by visual inspection of *de novo* transcripts (Manual) in human oocytes. Yellow: DMRs overlapped by a LIT in human oocytes, green: DMRs overlapped by a LIT in human oocytes showing evidence of paternal-specific transcription in the placenta (supplementary reference 10).
