# Supplementary material for: LTR retrotransposons transcribed in oocytes drive species-specific and heritable changes in DNA methylation
Source: Nat Commun. 2018 Aug 20;9:3331. doi: 10.1038/s41467-018-05841-x (PMC6102241; doi:10.1038/s41467-018-05841-x)
Supplement: Supplementary file 2 — Peer Review File [file 41467_2018_5841_MOESM2_ESM.pdf]

Editorial Note: this manuscript has been previously reviewed at another journal that is not operating a transparent peer review scheme. This document only contains reviewer comments and rebuttal letters for versions considered at Nature Communications.

REVIEWERS' COMMENTS:

Reviewer #1 (Remarks to the Author):

The revised paper is much improved and all my previous concerns have been addressed.

Reviewer #4 (Remarks to the Author):

The authors has sufficiently addressed the minor points raised in the first round of reviews, and I appreciate their good-faith effort to clarify issues in the rebuttal re: temporal establishment of H3K36me3 and DNA methylation in NGO/GO. I enthusiastically recommend publication and congratulate the authors on their solid work.
